# Investigating the Binding Mode of Reversible LSD1 Inhibitors Derived from Stilbene Derivatives by 3D-QSAR, Molecular Docking, and Molecular Dynamics Simulation

**DOI:** 10.3390/molecules24244479

**Published:** 2019-12-06

**Authors:** Yongtao Xu, Zihao He, Min Yang, Yunlong Gao, Linfeng Jin, Meiting Wang, Yichao Zheng, Xiaoyuan Lu, Songjie Zhang, Chang Wang, Zongya Zhao, Junqiang Zhao, Qinghe Gao, Yingchao Duan

**Affiliations:** 1 School of Medical Engineering, Xinxiang Medical University, Xinxiang 453003, China; hezihao311@163.com (Z.H.); yangmin@xxmu.edu.cn (M.Y.); zezt113919@163.com (Y.G.); wangmeitingbetter@163.com (M.W.); luxy@xxmu.edu.cn (X.L.); zsj2002@xxmu.edu.cn (S.Z.); wangchang@xxmu.edu.cn (C.W.); zhaozongya_paper@126.com (Z.Z.); 2 Xinxiang Key Laboratory of Biomedical Information Research, Xinxiang 453003, China; 3 Henan Engineering Laboratory of Combinatorial Technique for Clinical and Biomedical Big Data, Xinxiang 453003, China; 4 School of Pharmacy, Xinxiang Medical University, Xinxiang 453003, China; 5 State Key Laboratory of Precision Spectroscopy, School of Physics and Materials Science, East China Normal University, Shanghai 200062, China; 6 Key Laboratory of Advanced Pharmaceutical Technology, Ministry of Education of China, Co-Innovation Center of Henan Province for New Drug R & D and Preclinical Safety, Institute of Drug Discovery and Development, School of Pharmaceutical Sciences, Zhengzhou University, 100 Kexue Avenue, Zhengzhou 450001, China; yichaozheng@zzu.edu.cn; 7 College of Sanquan, Xinxiang Medical University, Xinxiang 453003, China; 06061002@sqmc.edu.cn

**Keywords:** LSD1, molecular inhibitors, stilbene derivatives, molecular docking, 3D-QSAR, molecular dynamics simulations

## Abstract

Overexpression of lysine specific demethylase 1 (LSD1) has been found in many cancers. New anticancer drugs targeting LSD1 have been designed. The research on irreversible LSD1 inhibitors has entered the clinical stage, while the research on reversible LSD1 inhibitors has progressed slowly so far. In this study, 41 stilbene derivatives were studied as reversible inhibitors by three-dimensional quantitative structure–activity relationship (3D-QSAR). Comparative molecular field analysis (CoMFA $q^{2}$ = 0.623, $r^{2}$ = 0.987, $r_{\mathrm{pred}}^{2}$ = 0.857) and comparative molecular similarity indices analysis (CoMSIA $q^{2}$ = 0.728, $r^{2}$ = 0.960, $r_{\mathrm{pred}}^{2}$ = 0.899) were used to establish the model, and the structure–activity relationship of the compounds was explained by the contour maps. The binding site was predicted by two different kinds of software, and the binding modes of the compounds were further explored. A series of key amino acids Val288, Ser289, Gly314, Thr624, Lys661 were found to play a key role in the activity of the compounds. Molecular dynamics (MD) simulations were carried out for compounds **04**, **17**, **21**, and **35**, which had different activities. The reasons for the activity differences were explained by the interaction between compounds and LSD1. The binding free energy was calculated by molecular mechanics generalized Born surface area (MM/GBSA). We hope that this research will provide valuable information for the design of new reversible LSD1 inhibitors in the future.

## 1. Introduction

Epigenetic post-transcriptional modifications of DNA or histone, such as methylation, acetylation, and phosphorylation, can remodel the chromatin structure (heterochromatin or euchromatin) to regulate the expression of genes [1,2]. Histone methylation has been considered irreversible. The discovery of lysine-specific demethylase 1 (LSD1, also known as KDM1A) in 2004 revealed that histone methylation was a dynamically adjustable process. LSD1 belongs to flavin adenine dinucleotide (FAD)-dependent amine oxidase family [3], and LSD1 functions as an enzyme that demethylates mono- and dimethylated histone 3 lysine 4 (H3K4me1, H3K4me2) and histone 3 lysine 9 (H3K9me1, H3K9me2) by utilizing its noncovalently bound FAD cofactor, which requires a pair of lone pairs of electrons in the substrate; therefore, LSD1 cannot act on trimethylated lysine [4]. LSD1 and RE1-silencing transcription factor corepressor 1 (CoREST) exist together in the form of transcriptional corepressor complexes. When LSD1 is combined with chromatin, CoREST can keep the overall structure of LSD1 stable [5]. LSD1 can also remove the methyl of nonhistone proteins, such as p53, signal transducer and activator of transcription 3 (STAT3), E2F transcription factor 1 (E2F1), and DNA methyltransferase 1 (DNMT1), which shows that LSD1 has an impact on the function of downstream cells [6,7,8,9,10]. The overexpression of LSD1 has been detected in various solid tumors, including retinoblastoma, non-small cell lung cancer, prostate cancer, breast cancer, acute myeloid leukemia, and colon cancer [11,12,13,14,15,16]. LSD1 is not only an important biological significance, but also a potential drug target for therapy of cancer.

**Figure 1:**
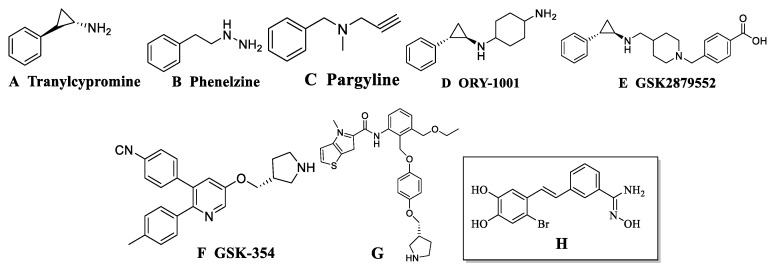
(**A**–**H**) represent structures of several reported lysine specific demethylase 1 (LSD1) inhibitors. Class H small molecules are the focus of this study.

Because LSD1 and monoamine oxidases (MAOs) A and B are homologous proteins, their sequence similarity reaches 17.6%. Some MAO inhibitors bond covalently with FAD to inhibit the activity of LSD1, such as tranylcypromine (TCPA), as seen in Figure 1A, phenelzine, as seen in Figure 1B, and pargyline, as seen in Figure 1C. Unfortunately, these inhibitors had no significant inhibitory effect on LSD1 and poor selectivity [17]. However, by optimizing the structure of these compounds, LSD1 inhibitors with high activity have been designed, such as ORY-1001, as seen in Figure 1D [18], and GSK2879552, as seen in Figure 1E [19], which are both TCPA derivatives. At present, they have entered clinical research and have shown good inhibition of LSD1: IC_50_ = 18 nM and IC_50_ = 20 nM, respectively. These inhibitors mentioned above were irreversible inhibitors, which were characterized by covalent binding with FAD. At the same time, many reversible LSD1 inhibitors have been reported, which can be divided into two categories: FAD-competing inhibitors and substrate-competing inhibitors. The IC_50_ of GSK-354, as seen in Figure 1F, was 90 nM [20]. In 2017, an inhibitor IC_50_ = 7.8 nM was reported and the structure was provided, as shown in Figure 1G [21]. In 2017 and 2018, a series of stilbene derivatives were reported and evaluated as potential inhibitors for the treatment of acute myelogenous leukemia (AML). The most active inhibitor IC_50_ = 121.23 nM is shown in Figure 1H [22,23]. Nowadays, there is no reversible inhibitor in clinical trials. Therefore, designing new efficient reversible inhibitors is still a big challenge.

In recent years, the rapid development of new drug research depends on computer-aided drug design (CADD) [24]. 3D-QSAR, molecular docking, and molecular dynamics (MD) are commonly used in CADD methods. 3D-QSAR can predict the activity of new compounds which contain a common skeleton. The key groups affecting the activity of compounds are found, which then provide guidance for the design of new compounds [25]. Molecular docking is often used to explore the binding mode of ligands and to study ligand–receptor interaction, but receptor flexibility cannot be determined during molecular docking [26]. However, in the process of MD, ligand and receptor flexibility can be taken into account, and the stability of the whole complex can also be observed [27]. Therefore, molecular docking and MD are usually combined to investigate possible bonding modes and detailed ligand–receptor interactions. At the same time, key amino acids which have important effects on activity will be found.

This study was mainly focused on the small molecule inhibitor of LSD1. It has been reported that stilbene derivatives have a significant effect on inhibiting LSD1 [22,23]. We hope to design and synthesize better LSD1 inhibitors on this basis. We synthesized 11 new stilbene derivatives and tested their activity using the same investigated enzyme inhibition experiment with LDS1 as the target. Unfortunately, their activity was not as good as we expected, even lower than the reported small molecules. In order to find out the reason for the difference of activity of these small molecules, we used CADD to carry out a series of studies. To explore the structure–activity relationship of these inhibitors, 41 complexes (30 collected from literature [22,23] and 11 newly synthesized) were used for building the 3D-QSAR model. In a series of stilbene derivatives reported in 2018, the optimal small molecule binding site in this series was the FAD cavity of LSD1, as determined by enzyme kinetics studies [23]. However, the binding site of a series of compounds reported in 2017 was not found. Because there are no reports on the crystal structures of such inhibitors, we combined molecular docking and MD to explore the possible binding mode of such compounds. We hope that a series of studies could find the key information affecting the activity of such inhibitors and help the development of reversible LSD1 inhibitors in the future.

## 2. Materials and Methods

### 2.1. Determination of LSD1 Inhibitor Activity

The IC_50_ testing of the 11 newly synthesized compounds was carried out in accordance with the previously reported method [22,23]. Synthetic routes and the characterization data of the compounds are shown in Supplementary Information S1. LSD1 demethylated its substrate H3K4me2 to form H3K4 and produced a molecule of hydrogen peroxide. In the presence of fluorescent dye Amplex Red and horseradish peroxidase (HRP), hydrogen peroxide can oxidize Amplex Red under the action of HRP to form a molecule of fluorescent Resorufin. Resorufin can be detected at an excitation wavelength of 530 nm and emits at 590 nm. When LSD1 inhibitors restrained the function of LSD1, the production of hydrogen peroxide decreased, and the production of Resorufin also decreased. Therefore, the inhibition ability of LSD1 could be judged by detecting the fluorescence intensity. LSD1 at a final concentration of 0.25 µM, and drugs with different concentrations were incubated at room temperature in HEPES buffer solution with final concentration of 50 mM pH = 7.5 for 10 min. Then the LSD1 substrate H3K4me2 at a final concentration of 25 µM (composed of **21** amino acids, polypeptide modified by the fourth lysine dimethyl group) was added and incubated at 37 °C for 10 min. Then, DMSO solution with a final concentration of 10 µM Amplex Red, and HEPES solution with 10 U/mL HRP were added and incubated at room temperature for 5 min. The fluorescence intensity was detected at 530 nm excitation wavelength and 590 nm emission wavelength by an enzyme labeling instrument.

The fluorescence intensity of the tested pore and 100% active pore were compared, and the inhibition rate of the compound at a specific concentration was calculated according to the following Equation (1).

(1) $$\mathrm{Inhibition}\left(\%\right)\;=\;\frac{\mathrm{Absorbance}\;\mathrm{of}\;\mathrm{control}-\mathrm{Absorbance}\;\mathrm{of}\;\mathrm{the}\;\mathrm{sample}}{\mathrm{Absorbance}\;\mathrm{of}\;\mathrm{the}\;\mathrm{control}}\times 100\%$$

All experiments were performed in triplicate.

### 2.2. Data Sets

The data set included 30 reported compounds [22,23] and 11 newly synthesized compounds. The structure and activity data of these 41 compounds are shown in Table 1. The 3D structure of all compounds was established by the Sketch module in SYBYL-X2.0. The range of pIC_50_ for data sets was from 4.443 to 6.917, the active span was large, and the distribution was uniform, which are the basic requirements of 3D-QSAR modeling. In the process of partitioning the training set and test set, the value of pIC_50_ should be satisfied: Max (test) ≤ max (training) and min (test) ≥ min (training) [28]. At the same time, the diversity of substituent groups of compounds in the test set should be considered. The distribution of pIC_50_ in the test set should be uniform in the whole data set, as seen in Figure S1. The number of test sets should be 20%–25% of the whole data set. Based on the above criteria, 32 training sets were used to build the 3D-QSAR model, and nine test sets (22% of the total data set) were used to test the predictive ability of the model.

### 2.3. Alignment and Generation of the 3D-QSAR Models

**Figure 2:**
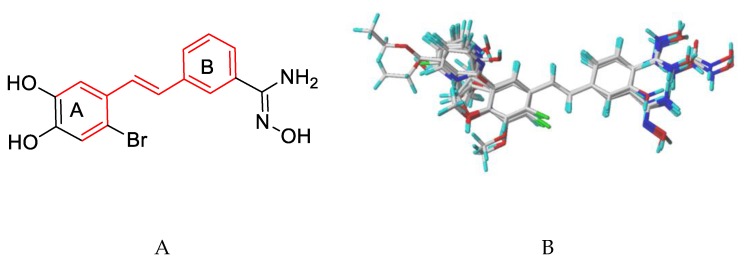
(**A**) Structure of compound **04**. The red color is the common framework for superposition. (**B**) Overlapping results of training set compounds.

The 3D-QSAR model was built using SYBYL-X2.0. Molecular alignment is generally considered to be a key factor affecting the stability and predictability of the model. This study used a common skeleton-based alignment approach. Firstly, Gastieger–Huckel charges were added to all small molecules to minimize energy under Tripos force field. Using the Powell gradient algorithm, the maximum number of iterations was set to 1000, and the convergence criterion was limited to 0.001 kcal mol^−1^ Å^−1^. Secondly, compound **04** with the highest activity, as seen in Figure 2A, was selected as the template molecule. Finally, the remaining small molecules were superimposed on the common skeleton of the red part of Figure 2A. The alignment results of the training set based on the common skeleton are shown in Figure 2B.

In this study, comparative molecular field analysis (CoMFA) and comparative molecular similarity indices analysis (CoMSIA) were used to establish 3D-QSAR models. CoMFA includes steric and electrostatic fields, which are calculated by Lennard-Jones and Coulombic potential functions. The superimposed molecule was placed in a three-dimensional space consisting of cube lattice (edge length = 2 Å). The structure of molecule was calculated by using SP^3^ hybrid carbon atom (van der Waals radius = 1.52 Å, net charge was + 1.0) as the probe atom. During the whole process, the energy cut-off value was set to 30 kcal/mol [29]. CoMSIA described the structural characteristics of molecule by calculating steric field, electrostatic field, hydrophobic field, hydrogen bond acceptor field, and hydrogen bond donor field parameters. The best CoMSIA model can be obtained by combining different fields. Like in CoMFA, a SP^3^ hybrid carbon atom is used as probe atom in the calculation of steric and electrostatic fields. Hydrophobic parameters, hydrogen bond acceptor parameters, and hydrogen bond donor parameters are set to +1 when calculating other fields. In the calculation process, the attenuation factor was set to 0.3. There is no need to set an energy cut-off value, because a Gaussian function is used to evaluate the distance between probe atom and each atom in molecule in CoMSIA [30]. As mentioned above, CoMSIA is an extension of CoMFA, and the principles are basically the same; both are forms of QSAR (quantitative structure analysis relationships). They differ only in the implementation of the fields. The following assumptions should be satisfied in application: 1) There is no covalent bond between the small molecule and the receptor; 2) The change of binding affinities of small molecules is related to molecular properties, represented by fields.

### 2.4. CoMFA and CoMSIA Statistical Analysis

Using partial least squares (PLS) regression analysis, we constructed linear a correlation between descriptors (independent variables) of the 3D-QSAR model and the pIC_50_ (dependent variables). Cross-validation correlation coefficient $q^{2}$ and optimum number of components (ONC) were obtained by leave-one-out (LOO) cross-validation [31]. $q^{2}$ is used to evaluate the internal validation ability of the model. Generally, $q^{2}$ > 0.5 is acceptable. The calculation equation(2) is as follows [32]:

(2) $$q^{2}\;=\;1-\frac{\sum {\left(y_{i}-y_{\mathrm{pred}}\right)}^{2}}{\sum {\left(y_{i}-\bar{y}\right)}^{2}}$$

where $y_{i}$ and $y_{\mathrm{pred}}$ represent the experimental and predicted values in the training set, respectively. $\bar{y}$ is the average value of the whole training set. Based on obtained ONC, the noncross-validation correlation coefficient $r^{2}$, F-statistic values (F), standard error of estimate (SEE), and the contribution of each field to the establishment of the model are further calculated by noncross-validation analysis. The external prediction ability of the model can be preliminarily evaluated by calculating the predictive correlation coefficient ($r_{\mathrm{pred}}^{2}$). $r_{\mathrm{pred}}^{2}$ > 0.6 means the model may have good prediction ability. The calculation equation(3) is as follows [25]:

(3) $$r_{\mathrm{pred}}^{2}\;=\;1-\frac{\mathrm{PRESS}}{\mathrm{SD}}$$

The PRESS represents the sum of squares of the difference between the experimental value and the predicted value in the test set. SD is the sum of squares of the difference between the experimental values of compounds in the test set and the average values in the training set.

However, $r_{\mathrm{pred}}^{2}>0.6$ is only the premise that the model has good external validation. The real external prediction ability needs evaluation of some external validation parameters, such as $R^{2}$, k, k′, $R_{0}^{2}$, $R{´}_{0}^{2}$, and $r_{m}^{2}$.${\;R}^{2}$ represents the correlation coefficients (not passing through the origin) between experimental values and the predicted values in the test set. $R_{0}^{2}$ and k are the correlation coefficients of the experimental value (X) and predicted value (Y) and the slope of regression line (passing through the origin). $R{´}_{0}^{2}$ and k′ are the correlation coefficients of the predicted value (Y) and experimental value (X) and the slope of regression line (passing through the origin). The calculation equations(4-9) are as follows [33]:

(4) $$R^{2}\;=\;\frac{{\left[\sum \left(Y_{\mathrm{obs}}-\bar{Y_{\mathrm{obs}}}\right)\left(Y_{\mathrm{pred}}-\bar{Y_{\mathrm{pred}}}\right)\right]}^{2}}{\sum {\left(Y_{\mathrm{pred}}-\bar{Y_{\mathrm{pred}}}\right)}^{2}\times \sum {\left(Y_{\mathrm{obs}}-\bar{Y_{\mathrm{obs}}}\right)}^{2}}$$

(5) $$k\;=\;\frac{\sum \left(Y_{\mathrm{obs}}\times Y_{\mathrm{pred}}\right)}{\sum {\left(Y_{\mathrm{pred}}\right)}^{2}}$$

(6) $$k´\;=\;\frac{\sum \left(Y_{\mathrm{obs}}\times Y_{\mathrm{pred}}\right)}{\sum {\left(Y_{\mathrm{obs}}\right)}^{2}}$$

(7) $$R_{0}^{2}\;=\;1-\frac{\sum {\left(Y_{\mathrm{obs}}-k\times Y_{\mathrm{pred}}\right)}^{2}}{\sum {\left(Y_{\mathrm{obs}}-\bar{Y_{\mathrm{obs}}}\right)}^{2}}$$

(8) $$R{´}_{0}^{2}\;=\;1-\frac{\sum {\left(Y_{\mathrm{pred}}-k´\times Y_{\mathrm{obs}}\right)}^{2}}{\sum {\left(Y_{\mathrm{pred}}-\bar{Y_{\mathrm{pred}}}\right)}^{2}}$$

(9) $$r_{m}^{2}\;={\;R}^{2}\times \left(1-\sqrt{R^{2}-R_{0}^{2}}\right)$$

In the above formulas,${\;Y}_{\mathrm{obs}}$ and $Y_{\mathrm{pred}}$ represent the experimental and predicted values in the test set.$\;\bar{Y_{\mathrm{obs}}}$ and $\bar{Y_{\mathrm{pred}}}$ are the average values of the experimental and predicted values in the test set.

The robustness of 3D-QSAR model can be verified by a Y-randomization test [34]. In the case of independent variable X, matrix unchanged, and randomly shuffled dependent variable Y, this process repeats many times, and new $q^{2}$ and $r^{2}$ values are recorded. If the values of $q^{2}$ and $r^{2}$ are very low, then the establishment of the model is not accidental and has strong robustness.

### 2.5. Molecular Docking

Before molecular docking, it is important to select the appropriate crystal structure. LSD1-CoREST complexes, including FAD and histone H3 (PDB ID: 2V1D, resolution: 3.1 Å), were used in this study. In order to obtain more reliable results, we chose MOE.2015 [35] and Glide of Maestro (Schr$\ddot{o}\mathrm{dinger}$ LLC, New York, NY, 2014-2) for docking. For Glide docking, firstly, we deleted crystal water from the PDB file and added hydrogen atoms to the entire complex. Then, we performed energy minimization. The stereochemical parameters of the model used for docking were evaluated using a Ramachandran plot and the overall goodness factor (G-factor) was obtained by Procheck [36]. In addition, verify 3D [37] and ERRAT [38] were used to evaluate the model (http://services.mbi.ucla.edu/saves/). Then, we used the prepared PDB file to generate the receptor-grid file. For the FAD site, we set FAD as the center and generated a box with side lengths of 20 Å × 20 Å × 20 Å. For substrate site, we set histone H3 as the center and generate a bo× with a side length of 20 Å × 20 Å × 20 Å. Finally, 41 small molecules after minimizing energy were docked to the FAD-binding site and substrate-binding site, separately. The standard precision mode (SP) was chosen, considering docking accuracy. Each small molecule was set to generate 20 poses, and the top ten poses by Glide score were saved for further study. The detailed process of MOE2015 is described in Supplementary Information S2.

### 2.6. Molecular Dynamics Simulations

In order to further explore ligand–receptor interaction and binding modes, 50 ns MD was performed on the docking results of compounds **04**, **17**, **21**, and **35**. MD was performed using AMBER 14 software package [39]. The antechamber module was used to generate ligand parameter files. Amberff10 force field was used for protein and GAFF force field was used for small molecules. The TIP3P water model was added and the margin was set to 8 Å. We checked the total charge of the whole system and added ${\mathrm{Cl}}^{-}$ to make the system appear electrically neutral. The topology file of the complex was generated in a water environment. After energy minimization, heating in an NVT ensemble (from 0 K to 300 K in 250 ps) and balancing 50 ps in an NPT ensemble (300 K, 1 atm) were carried out. Eventually, 50 ns MD was performed using the NPT ensemble (300 K, 1 atm).

### 2.7. Binding Free Energy Calculations

Binding affinity between small ligands and receptors can be evaluated by binding free energy. In this study, the binding free energy is calculated by the molecular mechanics/generalized Born surface area (MM/GBSA) method using AMBER 14 software. The structures were extracted from the last 2 ns trajectory file every 10 ps, and a total of 200 conformations were extracted for the calculation of binding free energy ($\Delta G_{bind}$). The calculation equations(10-12) are as follows [28]:

(10) $$\Delta G_{bind}\;=\;\Delta G_{complex}-\left(\Delta G_{protein}+\Delta G_{ligand}\right)$$

(11) $$=\;\Delta E_{gas}\;+\;\Delta G_{sol}\;-\;T\Delta S$$

(12) $$\Delta E_{gas}\;=\;\Delta E_{vdw}\;+\;\Delta E_{ele}\;\;\;\;\;\;\;\;\;\Delta G_{sol}\;=\;\Delta G_{GB}\;+\;\Delta G_{SA}$$

where $\Delta G_{\mathrm{complex}}$, $\Delta G_{\mathrm{protein}}$, and $\Delta G_{\mathrm{ligand}}$ represent the total binding energy of complex, protein, and ligand in solvent.$\;\Delta E_{\mathrm{gas}}$ is the interaction energy between the protein and ligand in gas phase, which can be further decomposed in to $\Delta E_{\mathrm{vdw}}$(van der Waals energy) and $\Delta E_{\mathrm{ele}}$(electrostatic energy).$\;\Delta G_{\mathrm{sol}}$ stands for free energy of solvation, and can be obtained by $\Delta G_{\mathrm{GB}}$(polar solvation energy) and $\Delta G_{\mathrm{SA}}$(nonpolar solvation energy). Then, $\Delta G_{\mathrm{GB}}$ is calculated by the generalized Born (GB) approximation model. TΔS is the entropy contribution. Because the calculation of this value is time-consuming and difficult to obtain accurately, the calculation of this term is often discarded [40].

## 3. Results and Discussion

### 3.1. Statistical Results of CoMFA and CoMSIA

To obtain the statistically best QSAR model, it is often necessary to adjust different field combinations (one or more) to establish multiple models, calculate their statistical parameters, and select the best CoMFA and CoMSIA models. The stepwise development [41] of the CoMFA model and several CoMSIA models using different combinations of fields are shown in Table 2. It is generally believed that the model with $q^{2}$ > 0.5 has good internal verification ability. The $q^{2}$ = 0.33 of CoMFA-E (only using electrostatic field to build the model) indicated that it did not have good internal verification ability. The $q^{2}$ = 0.547 and $r_{\mathrm{pred}}^{2}$ = 0.77 of CoMFA-S (only using stereo field to build models) showed that CoMFA-S had good internal verification and external prediction ability. However, the $q^{2}$ and $r_{\mathrm{pred}}^{2}$ of CoMFA-SE (combined with stereo and electrostatic fields) was considerably improved. The cross-validated coefficient $q^{2}$ = 0.623, and the predictive correlation coefficient $r_{\mathrm{pred}}^{2}$ = 0.857. The model also had larger noncross-validated coefficient $r^{2}$ = 0.987, lower SEE = 0.091, and F = 265.466. The contributions of electrostatic field and steric field were 38.6% and 61.4%, respectively. To sum up, CoMFA-SE was chosen as the final CoMFA model.

The $q^{2}$ of CoMSIA-SHAD and CoMSIA-SEHA were close, 0.728 and 0.726, respectively, indicating that they had good internal verification ability. However, the $r_{\mathrm{pred}}^{2}$ of CoMSIA-SHAD was higher than that of CoMSIA-SEHA (0.899 and 0.835, respectively), which indicated that CoMSIA-SHAD had stronger external prediction ability. The model had larger $r^{2}$ = 0.960, lower SEE value = 0.154, and F value = 126.052. The contributions of steric, hydrophobic, and H-bond acceptor and donor fields were 9.7%, 26.6%, 29.9%, and 33.9%, respectively. It showed that the H-bond donor field played an important role in this model. In conclusion, CoMSIA-SHAD was chosen as the final CoMSIA model.

Although $r_{\mathrm{pred}}^{2}$ > 0.6 for CoMFA-SE (hereafter referred to as CoMFA) and CoMSIA-SHAD (hereafter referred to as CoMSIA), according to Tropsha [42], good $r_{\mathrm{pred}}^{2}$ is only a prerequisite, and a series of external prediction parameters are needed to evaluate the true external prediction ability. The external predictive parameters of the model and the criteria to be met are shown in Table 3. When the model satisfied condition 1, condition 2a or 2b, condition 3a or 3b, condition 4a or 4b, condition 5, and condition 6, it can be evaluated that the model has strong real external prediction ability. $r_{m}^{2}$ denotes the approximation degree of the experimental and predicted values in the test set. CoMFA fit each criterion. CoMSIA did not meet condition 4b, but satisfied condition 4a and other conditions. Therefore, both CoMFA and CoMSIA had good external prediction ability.

**Figure 3:**
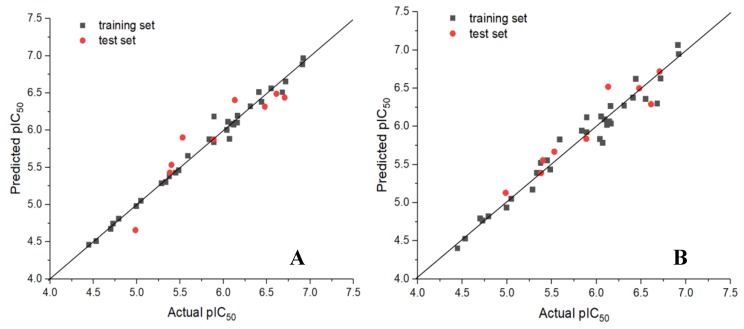
Plots of experimental activities against predicted activities by the optimal CoMFA model (**A**) and CoMSIA model (**B**).

The predicted values of CoMFA and CoMSIA models for training set and test set are shown in Table 1. The scatter plots of actual pIC_50_ and predicted pIC_50_ are shown in Figure 3. It can be seen from the figure that the black solid cubes and the red solid dots are close to the straight line Y = X. The actual value of the whole data set had a good linear relationship with the predicted value.

In addition, the robustness of the model was evaluated by Y-randomization testing. With the original independent variable X matrix unchanged, the dependent variable (pIC_50_) was randomly shuffled 10 times, and the details of randomly shuffled pIC_50_ values are shown in Table S1. If the $q^{2}$ and $r^{2}$ of these new models are very low or negative, it is not accidental that the final models have high $q^{2}$ and $r^{2}$. The Y-randomization test results of CoMFA and CoMSIA models are shown in Table 4. The $q^{2}$ and $r^{2}$ of the new model were very low, which showed that the previous final model has good robustness.

### 3.2. CoMFA Contour Maps

The 3D-QSAR model not only has the ability of prediction, but also can provide contour maps. It is more convenient to study the relationship between structure and activity of compounds to find the key groups affecting the activity, and to provide guidance for the design of new inhibitors in the future. The contour maps of each field are displayed using the StDev*Coeff function, and the visualization contribution of favorable and unfavorable regions is 80% and 20%, respectively. To explain the contour maps more clearly, the structure with the highest activity, compound **04**, was inserted into all contour maps.

**Figure 4:**
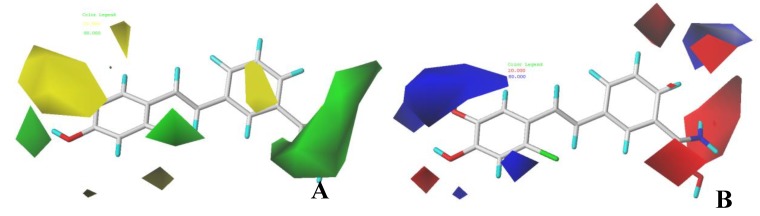
CoMFA contour maps were based on compound **04** as the reference. (**A**) Steric contour map. The green and yellow indicate that bulky groups are favored and disfavored, respectively. (**B**) Electrostatic contour map. The blue and red indicate that electropositive groups were favored and disfavored, respectively.

In the steric contour maps of CoMFA, the yellow block showed that reducing the volume of substituted groups contributed to the increase of activity, while the green color block showed that increasing the volume of the substituted groups was beneficial to the increase of activity. As shown in Figure 4A, a medium-sized green color block appeared at the R_2_ of compound **04**, suggesting that an appropriate addition of substituents here would be beneficial to the improvement of activity. For example, compounds **1** and **3** had hydroxyl substitution at R_1_, and compound **1** had a hydroxyl substitution at R_2_, while compound **3** had no hydroxyl substitution at R_2_ and a hydroxyl substitution at R_3_, so the activity of compound **1** (pIC_50_ = 6.478) was higher than that of compound **3** (pIC_50_ = 5.587). Compound **24** and compound **26** had methoxy substitution at R_1_, and compound **24** had methoxy at R_2_, but for compound **26**, there was no methoxy substitution at R_2_, methoxy substitution at R_3_, so the activity of **24** (pIC_50_ = 5.381) was higher than that of **26** (pIC_50_ = 5.285). The presence of a green block at R_4_ indicated that increasing the volume here would increase the activity; for example, the activities of compound **04** (R_4_ = Br, pIC_50_ = 6.917) and compound **05** (R_4_ = F, pIC_5_0 = 6.717) were higher than compound **1** (R_4_ = H, pIC_50_ = 6.478). Compound **10** (R_4_ = F, pIC_50_ = 6.706) and compound **11** (R_4_ = Br, pIC_50_ = 6.910) had higher activities than compound **7** (R_4_ = H, pIC_50_ = 6.131). A larger green block appeared at R_5_, indicating that the addition of larger substituents would improve the activity. For example, R_5_ of compound **13** (pIC_50_ = 5.373) was a hydroxyl group, while the R_5_ of compound **14** (pIC_50_ = 6.143) was an amidoxime, so the activity of compound **14** was higher than that of compound **13**. Compound **15** (pIC_50_ = 5.889) had amidoxime at R_5_, and compound **37** (pIC_50_ = 4.443) had hydroxyl at R_5_, so the activity of compound **15** was higher than that of compound **37**. A large yellow block appeared around R_1_, which indicated that the substituents in R_1_ should not be too large, otherwise the activity would be reduced. For example, the R_1_ of compound **2** (pIC_50_ = 6.611) was F, the R_1_ of compound **14** (pIC_50_ = 6.143) was pyridine, and the R_1_ of compound **15** (pIC_50_ = 5.889) was pyrimidine, so the activity of compound **2** was higher than that of compounds **14** and **15**. The R_1_ substituents of **39** (pIC_50_ = 4.696) and **40** (pIC_50_ = 4.722) were larger, so their activities were lower.

The contour maps of CoMFA electrostatic field are shown in Figure 4B. Blue indicated that the introduction of electropositive group was beneficial to the increase of activity, while the introduction of an electronegative group in a red region was favorable for increasing activity. There was a large blue block at R_1_, which indicated that the introduction of electropositive group could improve the activity. For example, the R_1_ of compound **18** (pIC_50_ = 6.066) was replaced by pyrimidine, the two N atoms of pyrimidine ring were covered by blue, and the R_1_ of compound **19** (pIC_50_ = 5.833) was replaced by hydroxyl pyridine, a strong electronegative group, so the activity of compound **18** was higher than compound **19**. There was a blue block at R_6_, which indicated that introducing a strong electropositive group here was beneficial to the increase of activity. The strong electronegative oxygen atom on the carbonyl of R_6_ of **25** (pIC_50_ = 5.480), **26** (pIC_50_ = 5.285), and **27** (pIC_50_ = 5.400) touched the blue block, so their activities were not high. There was a red block near R_5_, which indicated that the introduction of electronegative groups here would increase the activity. For example, R_5_ of compound **41** (pIC_50_ = 5.044) was replaced by –H, and its activity was low. R_5_ of compound 22 (pIC_50_ = 6.548) was replaced by amidoxime. The hydroxyl on amidoxime touched a red block, so the activity of compound **22** was higher than that of compound **41**. The R_5_ of compounds **4** (pIC_50_ = 6.917), **5** (pIC_50_ = 6.717), and **6** (pIC_50_ = 6.678) with higher activity were replaced by amidoxime, and the hydroxyl groups on the amidoxime were all near the red color block.

### 3.3. CoMSIA Contour Maps

**Figure 5:**
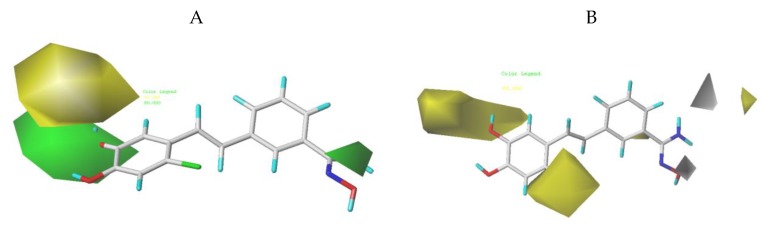

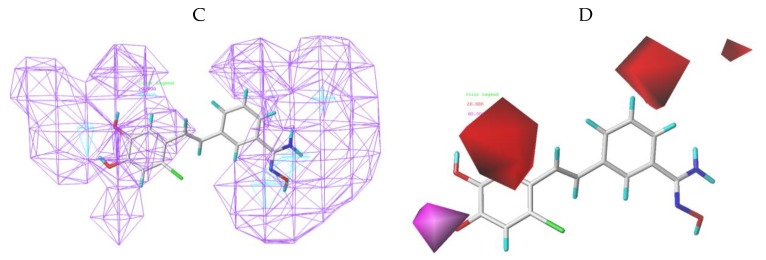
CoMSIA contour maps were based on compound **04** as a reference. (**A**) Steric field. The green and yellow blocks indicate that bulky groups are favored and disfavored, respectively. (**B**) Hydrophobic field. Yellow blocks indicate that hydrophobic groups increase activity; white blocks indicate that hydrophilic groups increase activity. (**C**) Hydrogen bond donor field (displayed as line). Cyan blocks indicate that H-bond donor groups increase activity; purple blocks indicate that H-bond donor groups decrease activity. (**D**) Hydrogen acceptor field. Magenta blocks indicate that H-bond acceptor groups increase activity; red blocks indicate that H-bond acceptor groups decrease activity.

The steric contour map of CoMSIA is shown in Figure 5A, which is similar to the conclusion of CoMFA: the proper increase of the volume of R_2_ and R_5_ substituents was beneficial to the increase of activity. The contour map of the CoMSIA hydrophobic field is shown in Figure 5B. The yellow part indicates that hydrophobic substituents would improve the activity, while the white area indicates that hydrophilic substituents would be beneficial to the activity. A yellow color block around R_1_ indicates that introducing hydrophobic substituents here is beneficial to the increase of activity; for example, compound **2** (pIC_50_ = 6.611, R_1_ = F) had higher activity than compound **1** (pIC_50_ = 6.478, R_1_ = OH). The R_1_ of compound **14** (pIC_50_ = 6.143) was a pyridine ring, and that of compound **15** (pIC_50_ = 5.889) was a pyrimidine ring, so the activity of compound **14** was higher than that of compound **15**. The presence of a yellow block at R_4_ indicated that the introduction of hydrophobic groups could improve the activity, such as **4** (pIC_50_ = 6.917, R_4_ = Br), **1** (pIC_50_ = 6.478, R_4_ = H), **25** (pIC_50_ = 5.480, R_4_ = F), **24** (pIC_50_ = 5.381, R_4_ = H). A small white block appeared near R_5_ and R_6_, which indicated that adding hydrophilic groups in these positions would be beneficial to increase activity. Hydroxyl and amino groups at the end of amidoxime are hydrophilic groups. Therefore, when amidoxime was substituted at R_5_ or R_6_, the activity was better, such as in compound **2** (pIC_50_ = 6.611) and compound **4** (pIC_50_ = 6.917), which had amidoxime structure at their R_5_ sites, and compound **11** (pIC_50_ = 6.910) and compound **16** (pIC_50_ = 6.036), which had amidoxime structures at R_6_.

As shown in Figure 5C, the CoMSIA hydrogen bond donor contour map showed that adding hydrogen bond donor groups in the cyan part was beneficial to improve the activity, while purple indicated that hydrogen bond donor groups in this position would hinder the activity. Because the cyan block was wrapped by the purple block, the display mode was set to line shape for convenience of observation. A cyan block at R_2_ indicated that introducing a hydrogen bond donor group at this position would be beneficial to improving activity. Compounds **5** (pIC_50_ = 6.717) and **14** (pIC_50_ = 6.143) had no hydrogen bond donor group at R_2_, so their activities were not high. There was a cyan block near R_5_, which indicated that introducing a hydrogen bond donor group at this position would be beneficial to the improvement of activity. Compounds **4** (pIC_50_ = 6.917), **22** (pIC_50_ = 6.548), and **6** (pIC_50_ = 6.678) with amidoxime structure are found at R_5_, so they had higher activity. The amino group in amidoxime just touched the cyan block, which may be the reason why compounds **4** and **6** had higher activity. The R_1_ and R_6_ were surrounded by a purple block, which indicated that introducing a hydrogen bond donor field into these positions was not conducive to the increase of activity. For example, compounds **35** (pIC_50_ = 4.790), **37** (pIC_50_ = 4.985), and **39** (pIC_50_ = 4.696) had H-bond donor groups at R_6_, while the substituent of R_1_ and R_6_ in compound **36** (pIC_50_ = 4.529) had H-bond donor groups. However, according to the H-bond donor field contour map, the presence of H-bond donor groups at R_1_ or R_6_ was not conducive to the activity of the compound, so their activities were low. These examples mean that if the positions of H-bond donor groups in the compounds conform to the positions given by the H-bond donor field contour map, the activities of these compounds were very high. On the contrary, if the H-bond donor groups appeared in the position which was not conducive to the activity suggested by the H-bond donor field contour map, the activity of these compounds would suddenly decline. These analyses explained why the contribution of H-bond donors was the highest in Table 2, because the H-bond donor group had an important influence on the activity of this series of compounds. As shown in Figure 5D, the magenta block indicated that the hydrogen bond receptor group had a positive effect on this position, while the red block indicated that the hydrogen bond receptor group had a negative effect on this position. One magenta block appeared at R_2_, suggesting that the addition of hydrogen bond receptor groups here would increase activity. For example, compound **26** (pIC_50_ = 5.285) had –H at R_2_, and compound **24** (pIC_50_ = 5.381) had a methoxy group at R_2_, so the activity of compound **24** was higher than that of compound **26**. A red color block appeared around R_6_, which indicated that adding a hydrogen bond acceptor group was not conducive to the improvement of activity. For example, the hydroxyl group at R_6_ of compound **12** (pIC_50_ = 4.991) and carbonyl group at R_6_ of compound **28** (pIC_50_ = 5.331) touched red blocks, so their activities were not high.

**Figure 6:**
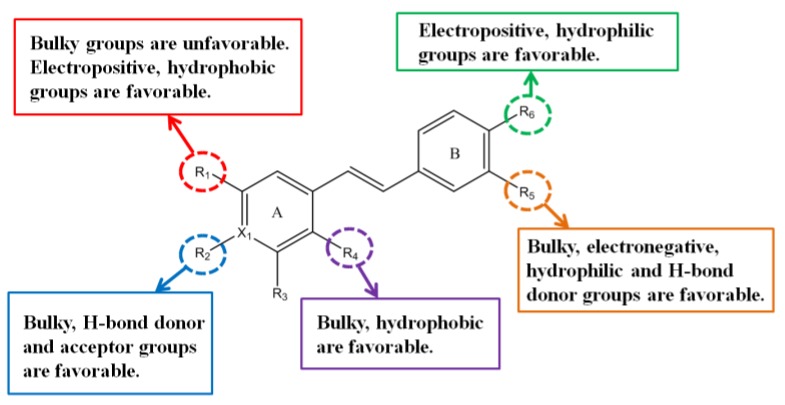
Structure–activity relationship (SAR) information obtained from three-dimensional quantitative structure–activity relationship (3D-QSAR) study.

The main structure–activity relationship (SAR) information found in 3D-QSAR is summarized in Figure 6. For R_1_, the electropositive groups with small volume and weak polarity should be introduced in the future compounds. If a larger volume group is introduced to R_2_, the activity might increase. It was found that H-bond donor groups and H-bond acceptor groups were encouraged at R_2_. Hydrogen and oxygen in hydroxyl groups can be used as H-bond donors and acceptors. Therefore, it is a good choice to introduce hydroxyl or carboxyl groups into R_2_. The optimization proposal of R_4_ is to introduce hydrophobic groups with large volume. In the existing compounds, R_4_ is generally replaced by a halogen. In the next design, we break this limitation and try esters and nitro structures. R_5_ should introduce large-volume, electronegative, hydrophilic H-bond donor groups. The amidoxime structure of this series of compounds can meet these requirements very well, so we can retain this structure in the future optimization, and try to introduce new structures, such as acylamide, carboxyl, etc. For R_6_, it is recommended to introduce electropositive and hydrophilic groups, and amino groups could be introduced. We hope that these suggestions for structural changes can provide new ideas for drug designers.

### 3.4. Bonding Site Prediction

**Figure 7:**
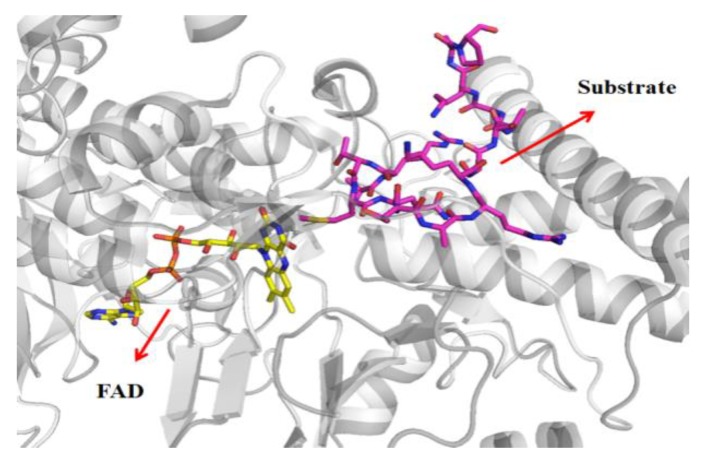
Binding site of flavin adenine dinucleotide (FAD) and histone H3 in LSD1.

Results and detailed analysis of the Ramachandran plot, verify 3D, and ERRAT used to test protein structure are shown in Supplementary Information S3. Enzyme kinetics studies were carried out on compound **22**, which is the most active of a series of compounds reported in 2018 [23]. It was proved that the binding site of these compounds was in the FAD region, while the binding sites of other compounds are not clear. Therefore, we need to determine the binding site in the FAD region or substrate region. The locations of these two regions are shown in Figure 7. Previous studies have shown that it is unreliable to evaluate binding affinity of ligand and receptor by docking score, and the correlation between them was very low [43]. However, it is reliable to predict the binding site of a small molecule by its docking score, and ligand scores at the correct binding site are better than those at the wrong site [44].

In order to get more credible results, Glide and MOE2015 were selected for the docking work. Before docking, FAD was extracted from the crystal structure (PDB: 2V1D), and then docked with two kinds of software to observe whether docking results can reproduce the crystal pose. When Glide was used, the root mean square deviation (RMSD) of the original crystal structure and docking result was 0.407 Å. When MOE2015 was used, the RMSD of the original crystal structure and docking result was 0.644 Å. Low RMSD indicated that the results were reasonable. The binding site of compound **22** in the FAD region justified by enzyme kinetics studies was selected as a reference. Compound **22** was docked in the FAD region and the substrate region using Glide and MOE2015, respectively. Among the remaining compounds, compound **04**, with the highest activity, was selected as the representative for binding site prediction. Similarly, compound **04** was docked in the FAD region and substrate region using two different kinds of software. Each docking result generated 20 poses, and the scores of the top five were recorded. The docking scores are shown in Table 5. For compound **22**, the top five scores of compound **22** in the FAD region and substrate region were −11.07 to −9.037 and −6.514 to −6.233, respectively, by Glide. The top five scores of compound **22** in the FAD region and substrate region by MOE2015 were −9.143 to −8.704 and −6.224 to −5.844, respectively. Both tools showed that compound **22** displayed lower scores in the FAD region. The results illustrated that the FAD region was more likely to be the binding site than the substrate region, which was also consistent with the results of enzyme kinetics studies. For Glide, the top five scoring ranges of compound **04** in the FAD region and substrate region were −9.132 to −8.724 and −6.364 to −5.315, respectively. For MOE2015, the top five scoring ranges of compound **04** in the FAD region and substrate region were −7.738 to −7.547 and −5.481 to −5.259, respectively. The two kinds of software showed that compound **04** scored lower in the FAD region. This indicated that for other compounds, the FAD region was more likely to be the binding site. This prediction was validated by calculating the binding free energy.

### 3.5. Exploration of Binding Mode

**Figure 8:**
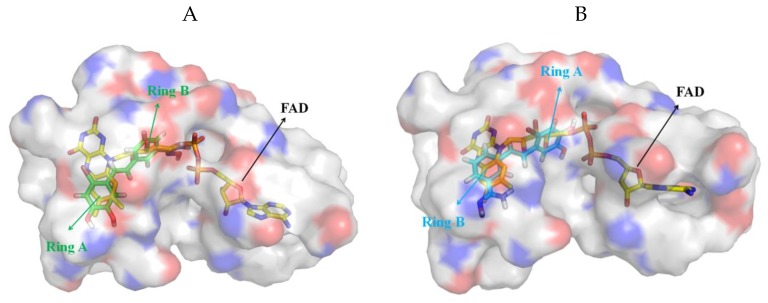
Two different binding orientations, with FAD shown as a yellow stick as a reference. (**A**) Compound Type A:04 represented by green sticks. (**B**) Compound Type B:04 represented by cyan sticks.

Based on the above analysis, all compounds were docked in the FAD region and their possible binding modes were explored. In order to increase the reliability of the results, two kinds of software were used. In all the docking results, there were mainly two opposite orientations, named type A and type B, respectively, as shown in Figure 8. This series of small molecules all contained ring A and ring B, as seen in Table 1. In order to be easy to express, compound **04** with the highest activity was displayed and FAD was used as a reference. Figure 8A shows the orientation of type A, and ring A of **04** overlapped with the triple-ring structure of FAD. Figure 8B shows the orientation of type B, and ring B of **04** overlapped with the triple-ring structure of FAD. Therefore, for all compounds, if ring A and the FAD triple-ring structure overlap, they were classified as type A, and if ring B and FAD triple-ring structure overlap, they were classified as type B.

In order to determine which orientation to use for the next study, the docking results of the top 10 for all compounds were recorded. The results of Glide, as shown in Table 6, recorded the times of occurrence of type A and type B, respectively, in the top 10, and recorded the highest scores belonging to type A or type B. The total number of type A and type B of some compounds was less than 10. This was because there were a few other orientations in the top 10, such as compounds **12** and **20**. However, these were rare orientations, so they were not recorded. In all 41 compounds, 31 compounds had more type A orientations in their top 10 conformations, and some compounds were all type A orientation in their top 10 conformations, such as compounds **13**, **16**, and **17**. There were two compounds in the top 10 with the same frequency of type A and type B. Only eight compounds in the top 10 showed more type B, but type A always appeared in the top 10. The top conformation of 30 compounds were in type A orientation; moreover, type B was not found in the top 10 conformations of 19 compounds. The docking results of compounds **04**, **05**, and **10** with high activity in type B are shown in Figure S5. These compounds did not even form H-bonds with surrounding amino acids, which was difficult to explain because they have high activity, indicating the irrationality of type B. It can be concluded that type A is the favored orientation of the compounds. Therefore, when choosing the docking results of all compounds for further research, one should ensure that the conformation with the best score belongs to type A orientation. The result of docking using MOE2015 is shown in Table S3. Therein, the top conformations of 30 compounds belonged to type A, the top conformations of five compounds belonged to neither type A nor type B, and only the top conformations of six compounds belonged to type B, which indicated that type A was most likely to be the true orientation of the compounds. 

The best docking structures of all compounds selected according to the above criteria are shown in Figure S6. They all entered FAD binding site well. The A ring was near the βsheet region, and the B ring was near the α helical region, and the linker in the middle was located in the coil region. However, by observing their overlap, it was found that although the orientations of these compounds were identical, there were still some differences in their binding modes.

**Figure 9:**
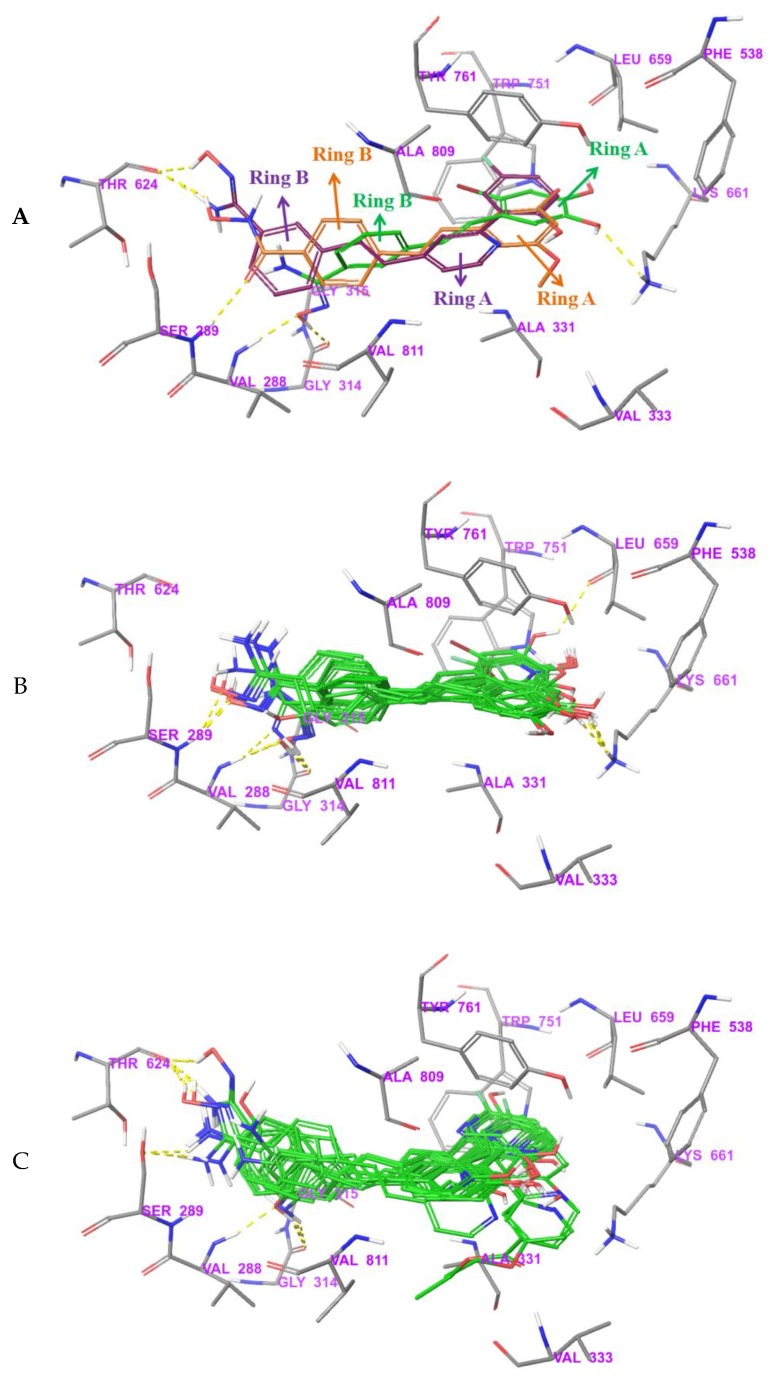

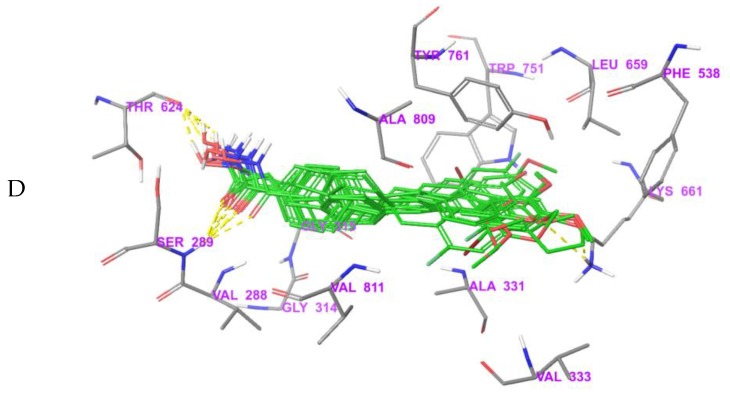
(**A**) Three binding modes for the three most active compounds; **04** is green, **22** is purple, and **29** is orange. (**B**) Superposition of 15 compounds in Category I. (**C**) Superposition of 15 compounds in Category II. (**D**) Superposition of 11 compounds in Category III.

According to the superposition of all small molecules, the binding modes of these compounds can be divided into three categories, expressed as I, II, and III, respectively. The categories of each compound are indicated in Table 1. Figure 9B–D show the binding modes of categories I, II, and III, respectively. For convenience of observation, the compounds with the highest activity (compounds **04**, **22**, and **29**) of the three binding modes are displayed in Figure 9A. The individual docking results of compounds **04**, **22**, and **29** are shown in Figure S7. Although their orientations were identical, the locations of ring A and ring B were different, which also led to the formation of different interactions between them and the protein. Ring A of compound **04** was surrounded by hydrophobic amino acids Phe538, Leu659, Trp751, Tyr761, and the B ring was surrounded by hydrophobic amino acids Val 288, Ala809, Val811. Oxygen and hydrogen on the amidoxime group formed H-bonds as H-bond acceptor and donor with Val288 and Gly314, respectively. In addition, oxygen on the hydroxyl of ring A formed an H-bond as a H-bond acceptor with Lys661. Compared with compound **04**, phenol attached to ring A of compound **22** and fluorine was added to para-position of the hydroxyl group. This new structure occupied the cavity formed by Phe538, Leu659, Trp751, and Tyr761, which resulted in ring A being squeezed into the vicinity of Ala331, Tyr761, and Ala809 in space. Thus, ring B also extended near Val288, Ser289, Gly315, and Thr624 and formed H-bonds with Thr624. The volume of ring A in compound **29** was larger than that of ring A of compound **04**. Moreover, the substitution position on ring B changed from the meta- to para-position, which indicated that more space was needed in the hydrophobic pocket. Finally, the positions of ring A and ring B changed a lot, and the carbonyl on R_6_ formed an H-bond as an H-bond acceptor with Ser289. Another H-bond was formed between Thr624 and the hydrogen of the hydroxyl group, which acted as an H-bond donor.

Based on the above analysis, 15 compounds with substituent volume of ring A similar to that of compound **04** and small substituent volume of ring B (e.g., amidoxime, hydroxyl) were classified as Category I, as shown in Figure 9B. These compounds were easily formed H-bonds with Val288, Ser289, Gly314, and Lys661. These amino acids may have an important effect on the activity. For example, both **03** and **04** formed H-bonds with Val288 and Gly314. The hydroxyl groups at R_2_ of **04** formed H-bonds with Lys661. There was no H-bond acceptor group at R_2_ of **03**, which may be the reason why the activity of **03** (pIC_50_ = 5.587) was lower than **04** (pIC_50_ = 6.917). Although the hydroxyl group at R_3_ of **12** formed an H-bond with Leu659, because of its long interaction distance (2.587 Å) and there were no other H-bonds, the activity was not high (pIC_50_ = 4.991). The meta-substitution of amidoxime at ring B usually formed two H-bonds with Val288 and Gly314, and the para-substitution of amidoxime at B ring formed one H-bond with Ser289. This explained why the meta-substitution of amidoxime at B ring was more active than the para-substitution of amidoxime at B ring; for example, compound **01** (meta-substitution of amidoxime at B ring) formed H-bonds with Val288 and Gly314, while compound **07** (para-substitution of amidoxime at B ring) did not form H-bonds with Val288 and Gly314, but only one H-bond with Ser289. Therefore, the activity of **01** (pIC_50_ = 6.478) was higher than that of **07** (pIC_50_ = 6.131). For the same reason, the activity of compound **2** (pIC_50_ = 6.611) was higher than that of compound **8** (pIC_50_ = 6.308). Compounds **35** (pIC_50_ = 4.790) and **37** (pIC_50_ = 4.985) did not form H-bonds with the surrounding amino acids, so their activity was very low.

Fifteen compounds with new ring structures attached to the A ring were classified as category II because they had similar interactions with proteins, as shown in Figure 9C. These compounds easily formed H-bonds with Ser289, Gly314, and Thr624. It was found that the activities of the compounds with para-substituted amino at B ring were not high. For example, compounds **17** (pIC_50_ = 5.447) and **23** (pIC_50_ = 5.529) had para-substituted amino groups on the B ring and formed only one H-bond with Ser289. Therefore, their activities were low. More hydrogen bonds were formed when the compounds were para-substituted at the B ring by amidoxime. For example, compounds **16** (pIC_50_ = 6.036) and **21** (pIC_50_ = 6.117) were para-substituted by aminoxime at ring B. Both of them formed H-bonds with Ser289 and Thr624, which may be the reason why their activities increased. Compounds **39** (pIC_50_ = 4.696) and **40** (pIC_50_ = 4.722) had low activities, because their newly introduced pyridine rings had extra structure, which was too large to enter the cavity formed by Phe538, Leu659, Trp751, and Tyr761. This led to the rotation of the rotational bonds between the pyridine rings and the A rings, and placed the pyridine near Ala331, Val333, and Lys661. The region was very close to the binding site, indicating that compounds **39** and **40** were not closely bound to proteins, which may be the reason for their poor activity. Therefore, when a new ring structure was introduced into the A ring, the addition of more groups in the new ring structure was not conducive to the improvement of activity.

Eleven compounds with larger substituted groups on the A ring than compound **04** and larger para groups at the B ring were classified as category III. As shown in Figure 9D, these compounds easily formed H-bonds with Ser289 and Thr624. Compared with category I, the substituents on the B ring of these compounds were larger, so they extended and formed H-bonds with Thr624. As H-bond acceptors and donors, carbonyl and hydroxyl groups in R_6_ were very important. For example, compound **32** (pIC_50_ = 6.154) not only formed H-bonds with Ser289 and Thr624, but also formed an H-bond with Lys611, so compound **32** had high activity. This was because the position of R_2_ was replaced by a methoxy group. Oxygen, as an H-bond acceptor, formed an H-bond with Lys611. Compound **28** (pIC_50_ = 5.331) formed H-bonds with Lys611, but not with Ser289 and Thr624, so its activity was low.

By observing the docking results, the R_5_ of compounds **1**, **2**, **4**, **5**, and **6** with high activity were aminoxime, and the hydroxyl, as an H-bond donor, formed H-bonds with Gly314. Similarly, R_5_ of compounds **20** and **22** with higher activity were replaced by aminoxime, in which the hydroxyl and amino groups formed H-bonds with Thr624 as H-bond donors. However, compounds **35** and **37** with lower activity did not have H-bond donor groups in R_5_, so they did not form H-bonds with the surrounding amino acids. This showed that for the possible H-bonds predicted in the H-bond donor field contour map, H-bonds were indeed formed in the molecular docking results, and the activities of these compounds were relatively high, while those compounds that did not form H-bonds at the predicted positions had relatively low activity. This evidence proves that H-bond donor groups have a great influence on the activity of this series of compounds from the perspective of molecular docking, and explains why H-bond donors had the highest contribution rate in the CoMSIA model.

### 3.6. MD Simulations and Binding Free Energy Calculation

**Figure 10:**
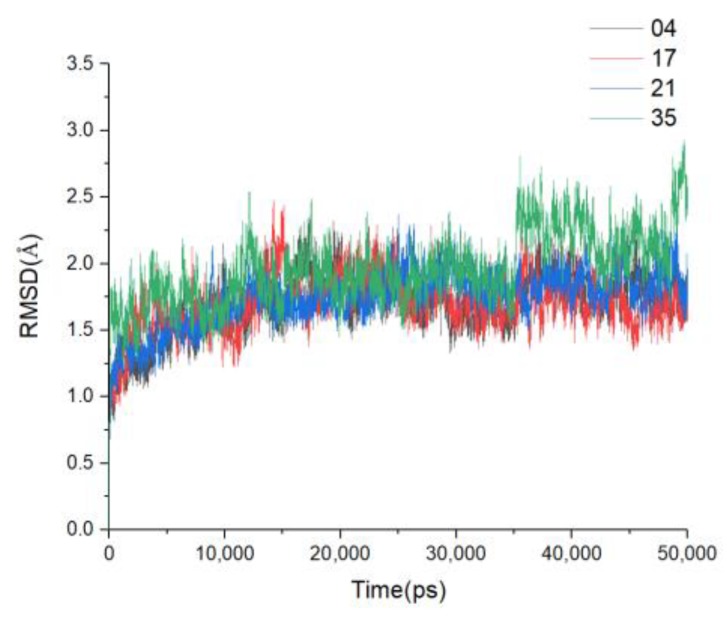
Root mean square deviation (RMSD) values of the complexes during 50 ns molecular dynamics (MD) simulations.

In order to further study the detailed interaction between ligands and receptor, and to verify the reliability of the results of molecular docking, compounds **04**, **17**, **21**, **35** with large activity range were selected for molecular dynamics simulation of 50 ns. The results of their docking were taken as the initial conformations. From the 50 ns MD process, the RMSD values for C_α_ of each complex are shown in Figure 10. After a short period of time, the four systems reached equilibrium. Compounds **04**, **17,** and **21** fluctuated in the range of 1.3–2.3 Å until 50 ns, while compound **35** fluctuated in the range of RMSD from 1.4 to 2.7 Å. The RMSD of compound **35** fluctuated around 36 ns. The superposition of the initial conformation of compound **35** and the conformation at 36 ns is shown in Figure S8. The surrounding residues only formed an H-bond (Glu801=O···HO, bond length 1.705Å) with the amidoxime connected to the B ring in the ligand, which might not be able to fix the position of the ligand, so the position of ring A changed greatly. At the same time, the polarity of compound **35** was strong, and there were polar amino acids around it, such as Thr, Ser, Asn, and Glu, which could also change of the position of the ligand. The activity of compound **35** was lower than that of the other three compounds, which could be the reason why the RMSD value of compound **35** fluctuated greatly. The temperature versus time plot is shown in Figure S9.

**Figure 11:**
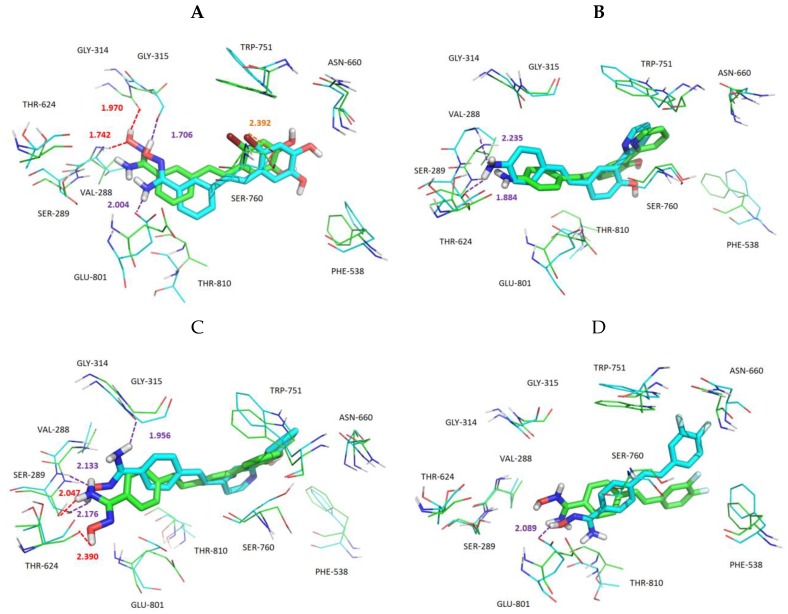
Superposition of the docking structures and MD average structures of compound **04** (**A**), **17** (**B**), **21** (**C**), and **35** (**D**). Carbon atoms of docking result and MD average structures are shown in green and cyan, respectively. H-bonds from docking and MD are shown as red dashed lines and purple dashed lines, respectively. Halogen bonds are represented by orange dotted lines.

As shown in Figure 11, for a clearer comparison of ligand–receptor interactions before and after MD, the docking conformations of compounds **04**, **17**, **21**, and **35**, and the corresponding average structure during the MD equilibrium stage were superimposed. The docking result of compound **04**, seen in Figure 11A, showed that the small molecule formed H-bonds with Val288 (Val288–NH···O, bond length 1.742 Å) and Gly314 (Gly314=O···HO, bond length 1.970 Å). Although these two H-bonds disappeared during the MD process, new H-bonds were formed with Gly315 (Gly315=O···HO, bond length 1.706 Å) and Glu801 (Glu801=O···HN, bond length 2.004 Å). In addition, Br on the A ring formed a halogen bond with Ser760 (bond length 2.392 Å). The docking result of compound **17**, seen in Figure 11B, showed that no H-bond was formed between the small molecule and the surrounding amino acids, while after MD, the result showed that compound **17** formed H-bonds with Val288 (Val288–NH···N, bond length 2.235 Å) and Ser289(Ser289–O···HN, bond length 1.884 Å). The docking result for compound **21**, as seen in Figure 11C, demonstrated that small molecule formed H-bonds with Ser289 (Ser289–O···HN, bond length 2.047Å) and Thr624 (Thr624=O···HO, bond length 2.390Å). During MD simulations, although compound **21** lost the H-bond with Thr624, it formed a shorter H-bond with Gly315 (Gly315 = O···HN, bond length 1.956Å). In addition, the hydroxyl of amidoxime, as an H-bond acceptor and donor, formed two H-bonds with Ser289. Compound **35** is shown in Figure 11D. The small molecule did not form any H-bonds with the surrounding amino acids, and only formed an H-bond with Glu801 (Glu801 = O···HO, bond length 2.089 Å) during the MD process. By MD analysis, compounds **17** and **35** formed fewer H-bonds and longer bond lengths, which indicated that the binding of these two small molecules with the protein was not close. However, compounds **04** and **21** formed more H-bonds and shorter bond lengths, and a halogen bond was formed between compound **04** and Ser760. This result demonstrated that the binding of compounds **04** and **21** with the protein was relatively stable.

From the above analysis, the binding affinity of compounds **04**, **17**, **21**, and **35** with LSD1 can be roughly judged. However, the binding affinity of small molecules with LSD1 was quantified by calculating the binding affinity. Therefore, the MM/GBSA method was used to calculate the binding free energy, as shown in Table 7.

The binding free energies of compounds **04**, **17**, **21**, and **35** with LSD1 were −41.196 kcal mol^−1^, −31.052 kcal mol^−1^, −35.516 kcal mol^−1^, and −28.063 kcal mol^−1^, respectively. The more negative the binding free energies of compounds, the better the experimental activity of the compounds. The van der Waals energy ΔE_vdw_ contributed most to the free energy, indicating that hydrophobic interaction played an important role in the binding process. In addition, electrostatic interaction ΔEele also contributed greatly to the binding free energy. The polar solvation energy ΔG_GB_ was positive, which indicated that it is disadvantageous to binding free energy, and the value of G_GB_ was larger. This may be due to the large binding pocket and the exposure of ligands to solvents. However, nonpolar solvation energy ΔG_SA_ was negative, which meant that it was good for binding free energy. Previous studies have shown that there is a significant correlation between binding free energy and experimental activity [45]. In this study, the binding free energies of the compounds were arranged in the same order as pIC_50_: **04** (pIC_50_ = 6.917) > **21** (pIC_50_ = 6.117) > **17** (pIC_50_ = 5.447) > **35** (pIC_50_ = 4.790). The binding free energy was calculated on the basis of the docking results. The pIC_50_ rank was consistent with the rank of binding free energy, which showed that the pose of ligands after docking was very close to the real binding mode, and verified that the previous selection of binding site was correct.

## 4. Conclusions

In this study, 41 stilbene derivatives were collected and synthesized, and a series of 3D-QSAR modeling, molecular docking, and molecular dynamics simulations were carried out. Firstly, CoMFA ($q^{2}$ = 0.623,$r_{\mathrm{pred}}^{2}$ = 0.857) and CoMSIA ($q^{2}$ = 0.728,$r_{\mathrm{pred}}^{2}$ = 0.899) models were constructed using the lowest energy conformation. The models had good internal verification ability and external prediction ability. In order to further evaluate the external prediction ability of the models, Tropsha and Roy criteria were used to evaluate the models. Statistically, our model was reliable and could be used to predict the unknown activity of stilbene derivatives to reduce experimental losses. At the same time, the contour maps were obtained, which reasonably explained the relationship between the structure and activity of the compounds, and summarized the key information. Secondly, the binding site of compounds was predicted by double software docking. On this basis, the binding modes of the compounds were explored and classified into three categories. Furthermore, the reasons for the differences in activity were explained. The hydrophobic amino acids Phe538, Leu659, Trp751, Tyr761, Ala809, and Val811 played important roles in the stability of small molecules in the binding pocket, and amino acids Val288, Ser289, Gly314, Thr624, and Lys661 played key roles in the formation of H-bonds. Finally, compounds **04**, **17**, **21**, and **35**, with a large activity range, were selected for MD. The interactions between those compounds and LSD1 were observed before and after MD. The reason for the poor activities of compounds **17** and **25** was explained. A halogen bond between compound **04** and Ser760 was also observed. The rank of binding free energies calculated by MM/GBSA coincided well with experimental activity, which indicated that the predictions of the binding site and binding mode were reasonable. We hope that this study will provide guidance and help for the design of new reversible LSD1 inhibitors.

## Figures and Tables

**Table 1:**
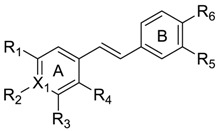
The structures and the actual and predicted activities of LSD1 inhibitors by comparative molecular field analysis (CoMFA) and comparative molecular similarity indices analysis (CoMSIA).

**No.**	**X_1_**	**R_1_**	**R_2_**	**R_3_**	**R_4_**	**R_5_**	**R_6_**	**IC_50_ (μM)**	**pIC_50_**	**CoMFA**		**CoMSIA**		**Binding Mode**
										**pred**	**res**	**pred**	**res**	
1 *	C	OH	OH	H	H	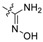	H	0.333	6.478	6.315	0.163	6.497	−0.019	I
2 *	C	F	OH	H	H	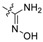	H	0.245	6.611	6.490	0.121	6.289	0.322	I
3	C	OH	H	OH	H	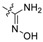	H	2.59	5.587	5.658	−0.071	5.831	−0.244	I
4	C	OH	OH	H	Br	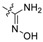	H	0.121	6.917	6.968	−0.051	6.95	−0.033	I
5	C	OH	OH	H	F	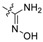	H	0.192	6.717	6.655	0.062	6.632	0.085	I
6	C	H	OH	H	H	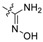	H	0.210	6.678	6.512	0.166	6.299	0.379	I
7 *	C	OH	OH	H	H	H	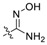	0.739	6.131	6.404	−0.273	6.518	−0.387	I
8	C	F	OH	H	H	H	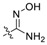	0.492	6.308	6.322	−0.014	6.275	0.033	I
9	C	H	OH	H	H	H	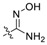	0.391	6.408	6.514	−0.106	6.38	0.028	I
10 *	C	OH	OH	H	F	H	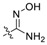	0.197	6.706	6.438	0.268	6.717	−0.011	I
11	C	OH	OH	H	Br	H	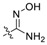	0.123	6.910	6.886	0.024	7.07	−0.160	I
12	C	OH	H	OH	H	H	OH	10.2	4.991	4.982	0.009	4.94	0.051	I
13	C	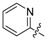	OH	H	H	OH	H	4.24	5.373	5.383	−0.010	5.526	−0.153	II
14	C	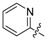	OH	H	H	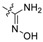	H	0.72	6.143	6.107	0.036	6.065	0.078	II
15	C	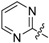	OH	H	H	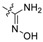	H	1.29	5.889	6.188	−0.299	6.121	−0.232	II
16	C	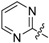	OH	H	H	H	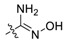	0.92	6.036	6.006	0.030	5.838	0.198	II
17	C	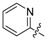	OH	H	H	H	NH_2_	3.57	5.447	5.432	0.015	5.557	−0.110	II
18	C	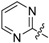	OH	H	H	NH_2_	H	0.859	6.066	5.884	0.182	5.788	0.278	II
19	C	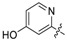	OH	H	H	NH_2_	H	1.47	5.833	5.88	−0.047	5.945	−0.112	II
20	N	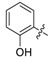	-	H	H	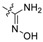	H	0.364	6.439	6.382	0.057	6.622	−0.183	II
21	N	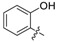	-	H	H	H	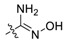	0.764	6.117	6.078	0.039	6.022	0.095	II
22	N	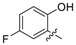	-	H	H	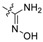	H	0.283	6.548	6.566	−0.018	6.361	0.187	II
23 *	N	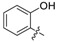	-	H	H	H	NH_2_	2.96	5.529	5.901	−0.372	5.667	−0.138	II
24 *	C	OMe	OMe	H	H	H	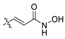	4.161	5.381	5.429	−0.048	5.388	−0.007	III
25	C	OMe	OMe	H	F	H	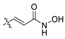	3.315	5.480	5.466	0.014	5.438	0.042	III
26	C	OMe	H	OMe	H	H	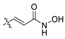	5.185	5.285	5.288	−0.003	5.176	0.109	III
27 *	C	OMe	OMe	H	Br	H	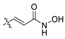	3.979	5.400	5.534	−0.134	5.553	−0.153	III
29	C	OMe	H	OMe	H	H	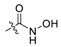	0.692	6.160	6.195	−0.035	6.038	0.122	III
30	C	OMe	OMe	H	H	H	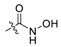	0.816	6.088	6.089	−0.001	6.095	−0.007	III
31 *	C	F	H	F	H	H	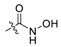	1.298	5.887	5.875	0.012	5.837	0.050	III
32	C	OMe	OMe	H	Br	H	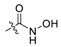	0.701	6.154	6.105	0.049	6.271	−0.117	III
34	C	OMe	OMe	H	F	H	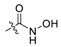	0.891	6.050	6.117	−0.067	6.13	−0.080	III
35	C	F	F	H	H	H	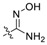	16.21	4.790	4.812	−0.022	4.825	−0.035	I
36	C	OH	OH	H	H	H	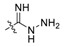	29.58	4.529	4.513	0.016	4.531	−0.002	I
38	C	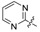	OH	H	H	OH	H	36.09	4.443	4.464	−0.021	4.405	0.038	II
39	C	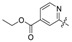	OH	H	H	-H	NH_2_	20.14	4.696	4.674	0.022	4.796	−0.100	II
40	C	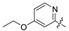	-	OH	H	NH_2_	H	18.96	4.722	4.747	−0.025	4.769	−0.047	II
41	N	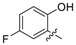	-	H	H	H	NH_2_	9.03	5.044	5.052	−0.008	5.052	−0.008	II
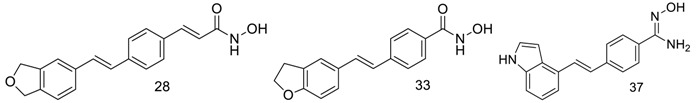														
			**No.**	**IC_50_ (μM)**			**pIC_50_**	**CoMFA**		**CoMSIA**		**Binding Mode**		
								**pred**	**res**	**pred**	**Res**			
			28	4.666			5.331	5.304	0.027	5.395	−0.0635	III		
			33	1.93			5.888	5.84	0.0482	5.927	−0.0386	III		
			37 *	10.36			4.985	4.657	0.3282	5.128	−0.1426	I		

* indicates the compound belongs to the test set. I, II, and III represent the binding modes of the compounds. Newly synthesized compounds have been underlined.

**Table 2:** Statistical parameters of CoMFA and CoMSIA models. S—Steric, E—Electrostatic, H—Hydrophobic, A—H-bond acceptor, D—H-bond donor.

	$q^{2}$	ONC	$r^{2}$	$r_{pred}^{2}$	SEE	F Value	Contributions				
							S	E	H	A	D
CoMFA-S	0.547	2	0.781	0.77	0.342	51.718	1	-	-	-	-
CoMFA-E	0.33	9	0.993	0.692	0.071	340.959	-	1	-	-	-
CoMFA-SE	0.623	7	0.987	0.857	0.091	265.466	0.386	0.614	-	-	-
CoMSIA-EHAD	0.674	5	0.962	0.800	0.151	129.944	-	0.276	0.214	0.226	0.283
CoMSIA-SHAD	0.728	5	0.960	0.899	0.154	126.052	0.097	-	0.266	0.299	0.339
CoMSIA-SEAD	0.639	4	0.942	0.803	0.182	110.417	0.094	0.326	-	0.254	0.325
CoMSIA-SEHD	0.700	4	0.937	0.819	0.190	100.367	0.090	0.329	0.239	-	0.342
CoMSIA-SEHA	0.726	6	0.977	0.835	0.120	174.365	0.098	0.325	0.258	0.319	-
CoMSIA-ALL	0.704	4	0.945	0.820	0.178	114.875	0.075	0.262	0.193	0.206	0.264

**Table 3:** Results of external validation parameters for CoMFA and CoMSIA.

Condition	Parameters	Threshold Value	CoMFA	CoMSIA
1	$R^{2}$	>0.6	0.855	0.861
2a	$R_{0}^{2}$	Close to value of R^2^	0.851	0.857
2b	$R{´}_{0}^{2}$	Close to value of R^2^	0.847	0.755
3a	k	0.85 < k < 1.15	1.001	0.983
3b	k´	0.85 < k` < 1.15	0.998	1.010
4a	$(R^{2}-R_{0}^{2})/R^{2}$	<0.1	0.005	0.005
4b	$(R^{2}-R{´}_{0}^{2})/R^{2}$	<0.1	0.009	0.123
5	$|R_{0}^{2}-R{`}_{0}^{2}|$	<0.3	0.004	0.102
6	$r_{m}^{2}$	>0.5	0.799	0.804

**Table 4:** q^2^ and r^2^ values after several Y-randomization tests.

	CoMFA		CoMSIA	
Iteration	$q^{2}$	$r^{2}$	$q^{2}$	$r^{2}$
Random_1	0.040	0.436	−0.052	0.466
Random_2	0.158	0.49	−0.003	0.435
Random_3	0.258	0.475	0.163	0.419
Random_4	0.086	0.401	0.071	0.35
Random_5	−0.113	0.484	−0.227	0.465
Random_6	0.262	0.484	0.317	0.505
Random_7	0.003	0.364	0.093	0.459
Random_8	−0.206	0.459	−0.271	0.497
Random_9	−0.131	0.423	−0.178	0.377
Random_10	−0.425	0.382	−0.627	0.454

**Table 5:** Docking scores of compounds **04** and **22** in the FAD and substrate regions calculated by Glide and MOE2015.

**Glide** **Top 5**	**FAD**		**Substrate**	
	**04**	**22**	**04**	**22**
1	−9.132	−11.070	−6.364	−6.514
2	−9.108	−9.503	−5.691	−6.406
3	−8.967	−9.125	−5.459	−6.249
4	−8.775	−9.102	−5.389	−6.234
5	−8.724	−9.037	−5.315	−6.233
**MOE2015** **Top 5**	**FAD**		**Substrate**	
	**04**	**22**	**04**	**22**
1	−7.738	−9.143	−5.481	−6.224
2	−7.680	−8.922	−5.475	−6.040
3	−7.611	−8.905	−5.434	−5.898
4	−7.571	−8.894	−5.341	−5.856
5	−7.547	−8.704	−5.259	−5.844

**Table 6:** Glide docking results. The second and third columns mean the number of type A and B in the top 10 conformations of all compounds. The last column includes the best scores and the type.

No.	Type A	Type B	Best Score
1	3	7	B −9.220
2	2	8	A −9.400
3	2	8	B −9.658
4	7	3	A −9.454
5	4	6	A −9.490
6	6	4	A −9.093
7	6	4	B −9.237
8	6	4	B −9.137
9	6	4	B −8.444
10	6	4	B −9.333
11	9	1	A −9.093
12	4	5	B −9.173
13	10	0	A −9.995
14	9	1	A −10.978
15	8	2	A −10.873
16	10	0	A −10.325
17	10	0	A −9.473
18	4	6	A −9.370
19	2	8	B −9.784
20	8	0	A −11.204
21	10	0	A −10.870
22	7	3	A −11.07
23	9	0	A −9.919
24	10	0	A −9.153
25	10	0	A −10.109
26	10	0	A −9.887
27	10	0	A −9.427
28	10	0	A −9.223
29	10	0	A −9.147
30	10	0	A −9.538
31	9	1	A −8.953
32	10	0	A −9.461
33	9	1	A −9.628
34	10	0	A −9.741
35	10	0	A −8.568
36	5	5	B −8.977
37	10	0	A −9.563
38	10	0	A −9.709
39	1	9	B −9.164
40	5	5	B −8.675
41	10	0	A −9.281

**Table 7:** Binding free energies of ligand–protein complexes.

No.	ΔE_ele_ kcal mol^−1^	ΔE_vdw_ kcal mol^−1^	ΔG_GB_ kcal mol^−1^	ΔG_SA_ kcal mol^−1^	ΔG_sol_ kcal mol^−1^	ΔG_bind_ kcal mol^−1^	pIC_50_
**LSD1-04**	−28.5803	−51.8392	45.2379	−6.0145	39.2235	−41.1960	6.917
**LSD1-17**	−14.5346	−45.4670	34.5108	−5.5613	28.9496	−31.0520	5.447
**LSD1-21**	−16.5911	−50.1944	37.4930	−6.2235	31.2696	−35.5160	6.117
**LSD1-35**	−15.5150	−38.2128	30.7264	−5.0611	25.6653	−28.0625	4.790

## Supplementary Materials

The following are available online, S1: Compound Characterization Data, Figure S1: 3D-QSAR Histogram of activity data distribution, S2: MOE2015 docking process, Table S1: Randomizations of biological activity for the Y-random test, S3: Structural validation, Figure S2: Ramachandran plot, Table S2: Residues falling in the core region of the Ramachandran’s plot, Figure S3: Verify 3D plots for model, Figure S4: ERRAT result for model, Figure S5: Docking results of the compound 04(A), 05(B) and 10(C) under type B, Table S3: MOE2015 docking results, Figure S6: All compounds were docked into the FAD-binding site, Figure S7: Docking results of the compound 04(A), 22(B) and 29(C) with LSD1, Figure S8: The superposition of the docking structure and structure of compound 35 in 36ns MD, Figure S9: Temperature fluctuation plot in MD.
