# Supplementary material for: Investigating the Binding Mode of Reversible LSD1 Inhibitors Derived from Stilbene Derivatives by 3D-QSAR, Molecular Docking, and Molecular Dynamics Simulation
Source: Molecules. 2019 Dec 6;24(24):4479. doi: 10.3390/molecules24244479 (PMC6943670; doi:10.3390/molecules24244479)
Supplement: Supplementary file 1 [file molecules-24-04479-s001.pdf]

Supplementary materials

Investigating the Binding Mode of Reversible LSD1 Inhibitors Derived from Stilbene derivatives by 3D-QSAR, Molecular Docking and Molecular Dynamics Simulation

Yongtao Xu[1,2,3,*], Zihao He[1,2,3], Min Yang[1,2,3],Yunlong Gao[1,2,3], Linfeng Jin[4], Meiting Wang[1,5], Yichao Zheng[6], , Xiaoyuan Lu[1], Songjie Zhang[1,2,3] , Chang Wang[1], Zongya Zhao[1], Junqiang Zhao[7] , Qinghe Gao[4*]and Yingchao Duan[4,*]

[1] School of Medical Engineering, Xinxiang Medical University, Xinxiang, Henan, 453003, China
[2] Xinxiang key laboratory of biomedical information research
[3] Henan engineering laboratory of combinatorial technique for clinical and biomedical big data
[4] School of Pharmacy, Xinxiang Medical University, Xinxiang, Henan, 453003, China
[5] State Key Laboratory of Precision Spectroscopy, School of Physics and Materials Science, East China Normal University, Shanghai，200062, China
[6] Key Laboratory of Advanced Pharmaceutical Technology, Ministry of Education of China, Co-innovation Center of Henan Province for New Drug R & D and Preclinical Safety, Institute of Drug Discovery and Development, School of Pharmaceutical Sciences, Zhengzhou University, 100 Kexue Avenue, Zhengzhou, Henan 450001, China
[7] College of Sanquan, Xinxiang Medical University, Xinxiang, Henan, 453003, China

* Correspondence: email:
yxu@xxmu.edu.cn (Y.X.); Tel.: +0086 15560156065;
gao_qinghe@xxmu.edu.cn(Q.G.); Tel.: +0086 15893849927;
duanyingchao1986@163.com(Y.D.); Tel: +0086 13781977941

S1. Compound Characterization Data

Reagents and solvents were purchased from commercial sources, when necessary, were purified and dried by standard methods. Melting points were determined on an X-5 micromelting apparatus and are uncorrected. ^{1}H NMR and ^{13}C NMR spectra were recorded on a Bruker Avance III HD 400 MHz and 100 MHz spectrometer at room temperature, using TMS as an internal standard. Chemical shifts were reported in ppm (d). Spin multiplicities were described as s (singlet), d (doublet), dd (double doublet), t (triplet), or m (multiplet). Coupling constants were reported in hertz (Hz). High resolution mass spectrometry (HRMS) was recorded on a Bruker MicrOTOF-Q III Micro mass spectrometer by electrospray ionization (ESI). Flash chromatography was performed on 200-300 mesh silica gel with the indicated solvent systems (Qingdao Haiyang Chemical, China).

General synthesis procedure for compounds **III** and **V**.

To a stirred solution of compound **I** (1.0 eq) and compound **II** or **IV** (1.05 eq) in dry DMF was added t-BuOK (3.0 eq) at 0℃. Then, the above reaction mixture was stirred for 0.5 h at room temperature. The mixture was poured into cold water, the resultant precipitate was filtered, washed with water, dried and purified by recrystallization from ethyl acetate to afford the pure product **III** or **V**.

General synthesis procedure for compounds **24-34**.

A solution of KOH (7.0 g, 127.3 mmol) in anhydrous methanol (20 ml) was added to a solution of hydroxylamine hydrochloride (5.84 g, 85.8 mmol) in anhydrous methanol (45 ml) at 0℃, and stirred for 5 mins and the white precipitate formed was filtered. The resultant filtrate (16 ml) was added to a solution of compound **III** or **V** (1.0 mmol) in anhydrous CH_2Cl_2 (5 ml) at 0℃, and then the mixture was stirred at room temperature for 1-2 h. The reaction mixture was evaporated under vacuum. The residue was acidified with 1 N HCl to a pH 5-6 and the resultant precipitate was filtered, washed with water, dried and purified by recrystallization from methanol to afford compound **24-34.**

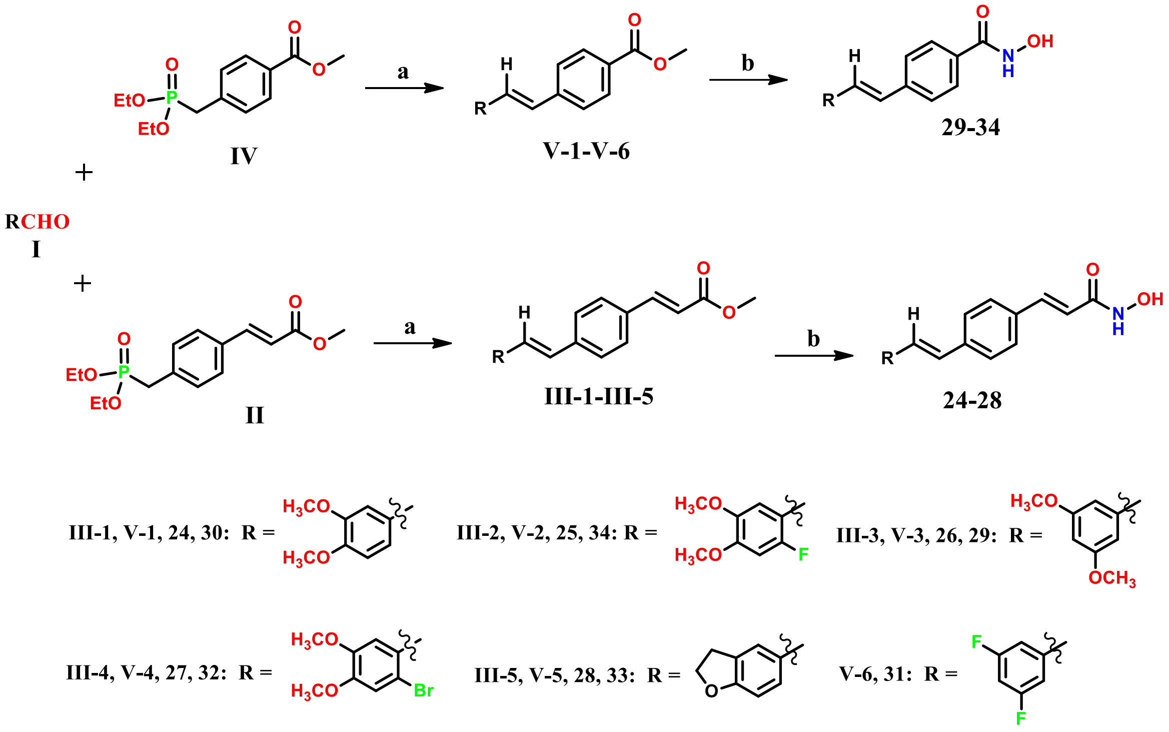

Scheme 1. Synthesis of compounds **24-34**. Reagents and conditions: (a) t-BuOK, anhydrous DMF, 0℃-rt, 0.5 h; (b) $NH_2OH{\cdot}HCl$, KOH, anhydrous CH_3OH-CH_2Cl_2, rt, 1-2 h.

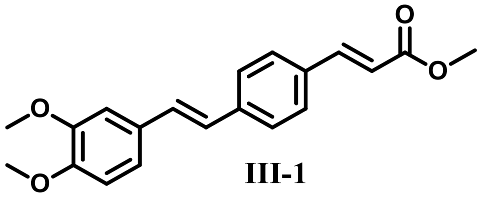

III-1

(E)-methyl 3-(4-((E)-3,4-dimethoxystyryl)phenyl)acrylate (**III-1**)

Yellow solid, Mp: 205-206℃, Yield: 71.3%. ^{1}H NMR (400 MHz, $CDCl_3$) δ 7.67 (d, 1H, *J* = 16.0 Hz), 7.53-7.50 (m, 4H), 7.12 (d, 1H, *J* = 16.4 Hz), 7.08-7.05 (m, 2H), 6.96 (d, 1H, *J* = 16.0 Hz), 6.87 (d, 1H, *J* = 8.4 Hz), 6.44 (d, 1H, *J* = 16.0 Hz), 3.95 (s, 3H), 3.91 (s, 3H), 3.81 (s, 3H). ^{13}C NMR (101 MHz, $CDCl_3$) δ 167.56, 149.33, 149.21, 144.42, 139.68, 133.25, 130.08, 129.96, 128.55, 126.67, 125.86, 120.29, 117.13, 111.25, 108.84, 55.97, 55.91, 51.71. HRMS (ESI) calcd for $C_{20}H_{20}NaO_4$ $[M + Na]^+$: 347.1254, Found: 347.1256.

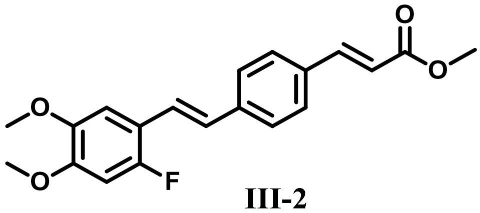

III-2

(E)-methyl 3-(4-((E)-2-fluoro-4,5-dimethoxystyryl)phenyl)acrylate (**III-2**)

Yellowish solid, Mp: 121-122℃, Yield: 62.9%. ^{1}H NMR (400 MHz, $CDCl_3$) δ 7.69 (d, 1H, *J* = 16.0 Hz), 7.54-7.49 (m, 4H), 7.28 (d, 1H, *J* = 16.8 Hz), 7.04 (d, 1H, *J* = 6.8 Hz), 7.00 (d, 1H, *J* = 16.4 Hz), 6.65 (d, 1H, *J* = 12.0 Hz), 6.44 (d, 1H, *J* = 16.0 Hz), 3.93 (s, 3H) , 3.89 (s, 3H) , 3.81 (s, 3H). ^{13}C NMR (101 MHz, $CDCl_3$) δ 156.41, 153.98,

149.97, 149.87, 145.58, 145.55, 144.36, 139.56, 133.50, 128.55, 127.46, 127.41, 126.80, 122.14, 122.11, 117.29, 115.95, 115.82, 108.33, 108.28, 100.29, 100.01, 56.45, 56.20, 51.72. HRMS (ESI) calcd for $C_{20}H_{19}NaO_4$ [M + Na]$^+$: 365.1160, Found :365.1162.

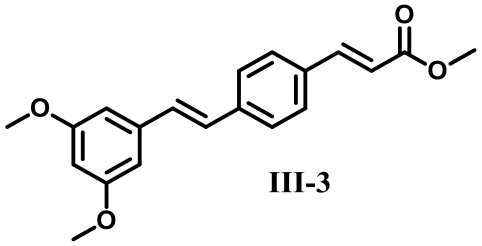

III-3

(E)-methyl 3-(4-((E)-3,5-dimethoxystyryl)phenyl)acrylate (**III-3**)
Yellow solid, Mp: 134-135℃, Yield: 70.4%. ^{1}H NMR (400 MHz, $CDCl_3$) δ 7.69 (d, 1H, *J* = 16.0 Hz), 7.53-7.50 (m, 4H), 7.11 (d, 1H, *J* = 16.4 Hz), 7.06 (d, 1H, *J* = 16.4 Hz), 6.68 (d, 2H, *J* = 2.0 Hz), 6.46-6.41 (m, 2H), 3.84 (s, 6H), 3.81 (s, 3H). ^{13}C NMR (101 MHz, $CDCl_3$) δ 167.52, 161.01, 144.33, 139.20, 138.93, 133.66, 130.08, 128.54, 128.26, 126.99, 117.39, 104.74, 100.34, 55.41, 51.75. HRMS (ESI) calcd for $C_{20}H_{20}NaO_4$ [M + Na]$^+$: 347.1254, Found: 347.1258.

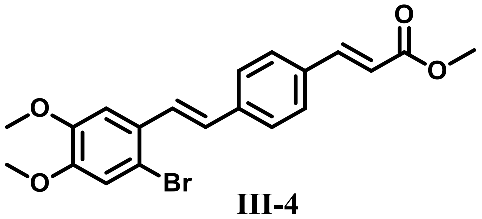

III-4

(E)-methyl 3-(4-((E)-2-bromo-4,5-dimethoxystyryl)phenyl)acrylate (**III-4**)
Yellow solid, Mp: 136-137℃, Yield: 66.2%. ^{1}H NMR (400 MHz, $CDCl_3$) δ 7.71 (d, 1H, *J* = 16.0 Hz), 7.57 (d, 2H, *J* = 8.8 Hz), 7.55 (d, 2H, *J* = 8.8 Hz), 7.48 (d, 1H, *J* = 16.4 Hz), 7.17 (s, 1H), 7.07 (s, 1H), 6.94 (d, 1H, *J* = 16.0 Hz), 6.47 (d, 1H, *J* = 16.0 Hz), 3.97 (s, 3H), 3.92 (s, 3H) , 3.84 (s, 3H). ^{13}C NMR (101 MHz, $CDCl_3$) δ 167.50, 149.67, 148.71, 144.32, 139.28, 133.71, 128.88, 128.70, 128.57, 128.43, 127.03, 117.44, 115.54, 115.31, 108.67, 56.22, 56.14, 51.73. HRMS (ESI) calcd for $C_{20}H_{19}BrNaO_4$ [M + Na]$^+$: 425.0359, Found: 425.0359.

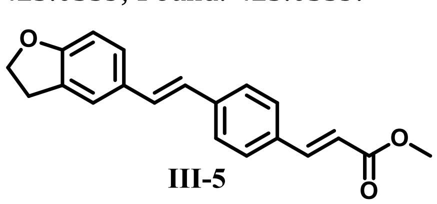

III-5

(E)-methyl 3-(4-((E)-2-(2,3-dihydrobenzofuran-5-yl)vinyl)phenyl)acrylate (**III-5**)
Yellow solid, Mp: 204-205℃, Yield 73.7%. ^{1}H NMR (400 MHz, $CDCl_3$) δ 7.68 (d, 1H, *J* = 16.0 Hz), 7.50 (d, 2H, *J* = 8.8 Hz), 7.47 (d, 2H, *J* = 8.8 Hz),7.41 (s, 1H) , 7.26 (dd, 1H, J_1 = 1.2 Hz, J_2 = 7.6 Hz), 7.11 (d, 1H, *J* = 16.4 Hz), 6.92 (d, 1H, *J* = 16.4 Hz), 6.78 (d, 1H, *J* = 8.4 Hz), 6.42 (d, 1H, *J* = 15.6 Hz), 4.60 (t, 2H, *J* = 8.4 Hz), 3.81 (s, 3H) , 3.23 (t, 2H, *J* = 8.8 Hz). ^{13}C NMR (101 MHz, $CDCl_3$) δ 167.60, 160.40, 144.49, 139.94, 133.02, 130.15, 129.85, 128.55, 127.79, 127.52, 126.57, 125.02, 122.88, 116.96, 109.51, 71.59, 51.71, 29.56. HRMS (ESI) calcd for $C_{20}H_{18}NaO_3$ [M + Na]$^+$: 329.1148, Found:

329.1146.

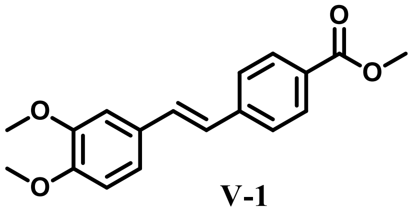

V-1

(E)-methyl 4-(3,4-dimethoxystyryl)benzoate (**V-1**)

White solid, Mp: 132-133℃, Yield: 71.1%. ^{1}H NMR (400 MHz, $CDCl_3$) δ 8.0 (d, 2H, *J* =8.4 Hz), 7.53 (d, 2H, *J* = 8.4 Hz), 7.15 (d, 1H, *J* =16.4 Hz), 7.08-7.05 (m, 2H), 6.98 (d, 1H, *J* = 16.4 Hz), 6.86 (d, 1H, *J* = 8.4 Hz), 3.94 (s, 3H), 3.91 (s, 3H) , 3.90 (s, 3H). ^{13}C NMR (101 MHz, $CDCl_3$) δ 166.94, 149.44, 149.17, 142.08, 131.04, 130.04, 129.84, 128.53, 126.06, 125.61, 120.46, 111.19, 108.83, 55.95, 55.89, 52.08. HRMS (ESI) calcd for $C_{18}H_{18}NaO_4$ $[M + Na]^+$: 321.1097, Found: 321.1094.

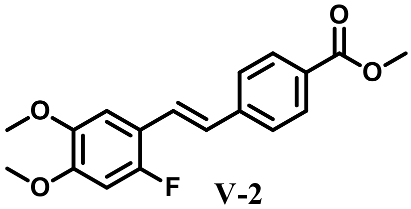

V-2

(E)-methyl 4-(2-fluoro-4,5-dimethoxystyryl)benzoate (**V-2**)

White solid, Mp: 102-104℃, Yield: 80.8%. ^{1}H NMR (400 MHz, $CDCl_3$) δ 8.02 (d, 2H, *J* = 8.4 Hz), 7.56 (d, 2H, *J* = 8.0 Hz), 7.32 (d, 1H, *J* = 16.4 Hz), 7.06-7.00 (m, 2H), 6.65 (dd, 1H, J_1 = 3.6 Hz, J_2 = 12.0 Hz), 3.93 (s, 3H), 3.92 (s, 3H), 3.89 (s, 3H). ^{13}C NMR (101 MHz, $CDCl_3$) δ 166.90, 156.51, 154.08, 150.13, 150.03, 145.58, 145.56, 141.96, 130.04, 128.81, 127.26, 127.22, 126.19, 123.26, 123.23, 115.74, 115.61, 108.37, 108.32, 100.29, 100.00, 56.44, 56.20, 52.09. HRMS (ESI) calcd for $C_{18}H_{17}FNaO_4$ $[M + Na]^+$: 339.1003, Found: 339.1008.

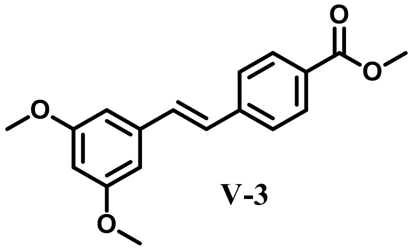

V-3

(E)-methyl 4-(3,5-dimethoxystyryl)benzoate (**V-3**)

White solid, Mp: 116-117℃, Yield: 75.9%. ^{1}H NMR (400 MHz, $CDCl_3$) δ 8.02 (d, 2H, *J* = 8.4 Hz), 7.55 (d, 2H, *J* = 8.4 Hz), 7.12 (d, 1H, *J* = 16.4 Hz), 7.09 (d, 1H, *J* = 16.4 Hz), 6.69 (d, 2H, *J* = 2.4 Hz), 6.43 (t, 1H, *J* = 2.0 Hz), 3.92 (s, 3H), 3.83 (s, 6H). ^{13}C NMR (101 MHz, $CDCl_3$) δ 166.88, 161.04, 141.64, 138.75, 131.22, 130.04, 129.01, 128.08, 126.40, 104.86, 100.56, 55.41, 52.11. HRMS (ESI) calcd for $C_{18}H_{18}NaO_4$ $[M + Na]^+$: 321.1097, Found: 321.1096.

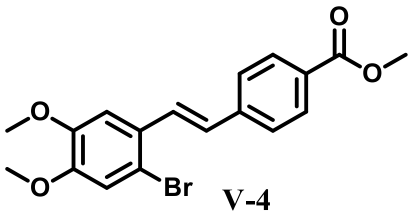

V-4

(E)-methyl 4-(2-bromo-4,5-dimethoxystyryl)benzoate (**V-4**)
White solid, Mp: 125-126℃, Yield: 64.6%. ^{1}H NMR (400 MHz, $CDCl_3$) δ 8.03 (d, 2H, *J* = 8.4 Hz), 7.58 (d, 2H, *J* = 8.4 Hz), 7.50 (d, 1H, *J* = 16.4 Hz), 7.15 (s, 1H), 7.05 (s, 1H), 6.94 (d, 1H, *J* = 16.0 Hz), 3.95 (s, 3H), 3.93 (s, 3H), 3.90 (s, 3H). ^{13}C NMR (101 MHz, $CDCl_3$) δ 166.86, 149.80, 148.69, 141.66, 130.07, 129.75, 129.02, 128.67, 128.23, 126.43, 115.51, 115.45, 108.69, 56.21, 56.13, 52.12. HRMS (ESI) calcd for $C_{18}H_{17}BrNaO_4$ [M + Na]$^+$: 399.0202, Found: 399.0207.

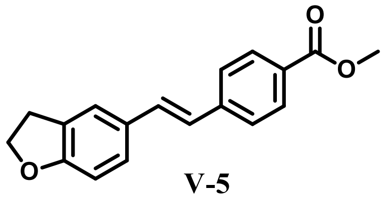

V-5

(E)-methyl 4-(2-(2,3-dihydrobenzofuran-5-yl)vinyl)benzoate (**V-5**)
White solid, Mp: 180-181℃, Yield: 72.6%.^{1}H NMR(400 MHz, $CDCl_3$) δ 8.00 (d, 2H, *J* = 8.4 Hz), 7.51 (d, 2H, *J* = 8.4 Hz), 7.41 (s, 1H), 7.27 (dd, 1H, J_1 = 2.0 Hz, J_2 = 7.6 Hz), 7.15 (d, 1H, *J* = 16.4 Hz), 6.95 (d, 1H, *J* = 16.4 Hz), 6.78 (d, 1H, *J* = 8.4 Hz), 4.60 (t, 2H, *J* = 8.8 Hz), 3.91 (s, 3H), 3.23 (t, 2H, *J* = 8.8 Hz). ^{13}C NMR (101 MHz, $CDCl_3$) δ 166.98, 160.55, 142.35, 131.24, 130.03, 129.66, 128.35, 127.82, 127.67, 125.96, 124.79, 123.00, 109.53, 71.61, 52.06, 29.55. HRMS (ESI) calcd for $C_{18}H_{16}NaO_3$ [M + Na]$^+$: 303.0992, Found: 303.0993.

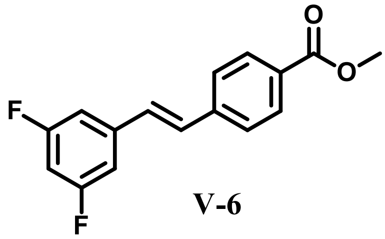

V-6

(E)-methyl 4-(3,5-difluorostyryl)benzoate (**V-6**)
White solid, Mp: 116-117℃, Yield: 59.5%. ^{1}H NMR (400 MHz, $CDCl_3$) δ 8.03 (d, 2H, *J* = 8.4 Hz), 7.54 (d, 2H, *J* = 8.4 Hz), 7.10-7.07 (m, 2H), 7.04-6.99 (m, 2H), 6.75-6.69 (m, 1H), 3.92 (s, 3H). ^{13}C NMR (101 MHz, $CDCl_3$) δ 166.71, 164.60, 164.47, 162.13, 162.00, 140.71, 140.25, 140.15, 140.06, 130.11, 129.66, 128.93, 128.90, 128.87, 126.64, 109.48, 109.41, 109.29, 109.22, 103.59, 103.34, 103.08, 52.17. HRMS (ESI) calcd for $C_{16}H_{12}F_2NaO_2$ [M + Na]$^+$: 297.0698, Found: 297.0697.

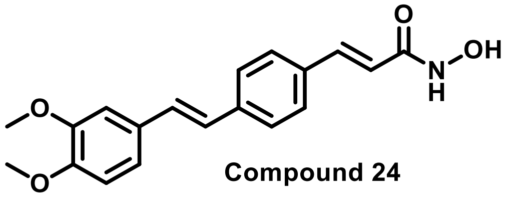

Compound 24

(E)-3-(4-((E)-3,4-dimethoxystyryl)phenyl)-N-hydroxyacrylamide (**24**)

Yellow solid, Mp:205-206℃, Yield: 55.4%. ^{1}H NMR (400 MHz, DMSO-d_6) δ 10.75 (s, 1H), 9.05 (s, 1H), 7.61 (d, 2H, *J* = 8.0 Hz), 7.55 (d, 2H, *J* = 8.0 Hz), 7.46 (d, 1H, *J* =15.6 Hz), 7.28-7.24 (m, 2H), 7.18-7.11 (m, 2H), 6.96 (d, 1H, *J* = 8.4 Hz), 6.47 (d, 1H, *J* = 15.6 Hz), 3.83 (s, 3H), 3.78 (s, 3H). ^{13}C NMR (101 MHz, DMSO-d_6) δ 163.35, 149.43, 139.12, 138.44, 134.03, 130.30, 129.91, 128.41, 127.07, 126.06, 120.74, 118.93, 112.24, 109.73, 55.98. HRMS (ESI) calcd for $C_{19}H_{18}NO_4$ [M－H]$^-$:324.1241, Found: 324.1235.

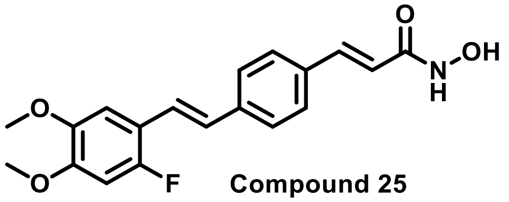

Compound 25

(E)-3-(4-((E)-2-fluoro-4,5-dimethoxystyryl)phenyl)-N-hydroxyacrylamide (**25**)

Yellow solid, Mp:185-186℃, Yield: 59.1%. ^{1}H NMR (400 MHz, DMSO-d_6) δ 10.77 (s, 1H), 9.07 (s, 1H), 7.63 (d, 2H, *J* = 8.4 Hz), 7.57 (d, 2H, *J* = 8.4 Hz), 7.46 (d, 1H, *J* = 16.0 Hz), 7.33-7.27 (m, 3H), 6.94 (d, 1H, *J* = 12.0 Hz), 6.48 (d, 1H, *J* = 16.0 Hz), 3.83 (s, 3H), 3.80 (s, 3H). HRMS (ESI) calcd for $C_{19}H_{18}FNNaO_4$ [M + Na]$^+$: 366.1112, Found: 366.1112.

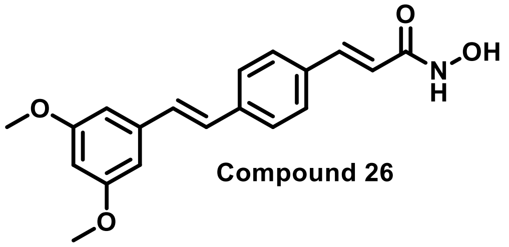

Compound 26

(E)-3-(4-((E)-3,5-dimethoxystyryl)phenyl)-N-hydroxyacrylamide (**26**)

Yellowish solid, Mp:149-150℃, Yield: 48.9%. ^{1}H NMR (400 MHz, DMSO-d_6) δ 10.76 (s, 1H), 9.06 (s, 1H), 7.65 (d, 2H, *J* = 8.0 Hz), 7.57 (d, 2H, *J* = 8.0 Hz), 7.46 (d, 1H, *J* = 15.6 Hz), 7.31 (d, 1H, *J* = 16.4 Hz), 7.25 (d, 1H, *J* = 16.4 Hz), 6.81 (d, 2H, *J* = 1.2 Hz), 6.48 (d, 1H, *J* = 15.6 Hz), 6.50-6.43 (m, 2H), 3.79 (s, 6H).^{13}C NMR (101 MHz, DMSO-d_6) δ 163.28, 161.15, 139.39, 138.59, 138.36, 134.58, 129.87, 128.76, 128.41, 127.47, 119.27, 105.10, 100.62, 55.71. HRMS (ESI) calcd for $C_{19}H_{19}NNaO_4$ [M + Na]$^+$: 348.1206, Found: 348.1207.

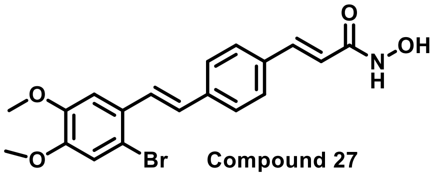

Compound 27

(E)-3-(4-((E)-2-bromo-4,5-dimethoxystyryl)phenyl)-N-hydroxyacrylamide (**27**)
Yellow solid, Mp:120-121℃, Yield: 62.5%. ^{1}H NMR (400 MHz, DMSO-d_6) δ 10.76 (s, 1H), 9.07 (s, 1H), 7.63 (d, 2H, *J* = 8.0 Hz), 7.59 (d, 2H, *J* = 8.0 Hz), 7.49-7.34 (m, 3H), 7.28 (d, 1H, *J* =16.0 Hz), 7.19 (s, 1H), 6.47 (d, 1H, *J* = 16.0 Hz), 3.86 (s, 3H), 3.81 (s, 3H). HRMS (ESI) calcd for $C_{19}H_{18}BrNNaO_4$ $[M + Na]^+$: 426.0311, Found: 426.0313.

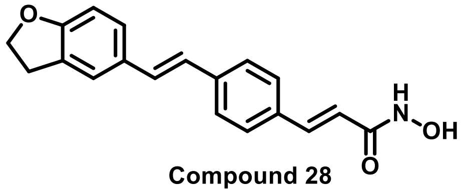

Compound 28

(E)-3-(4-((E)-2-(2,3-dihydrobenzofuran-5-yl)vinyl)phenyl)-N-hydroxyacrylamide (**28**)
Yellow solid, Mp:178-179℃, Yield: 51.8%. ^{1}H NMR (400 MHz, DMSO-d_6) δ 10.76 (s, 1H), 9.07 (s, 1H), 7.60 (d, 2H, *J* = 8.4 Hz), 7.55 (d, 2H, *J* = 8.0 Hz), 7.46 (d, 1H, *J* = 15.6 Hz), 7.38-7.32 (m, 2H), 7.25 (d, 1H, *J* = 16.4 Hz), 7.16 (d, 1H, *J* = 7.2 Hz), 6.86 (t, 1H, *J* = 7.6 Hz), 6.47 (d, 1H, *J* = 16.0 Hz), 4.60 (t, 2H, *J* = 8.8 Hz), 3.21 (t, 2H, *J* = 8.8 Hz). ^{13}C NMR (101 MHz, DMSO-d_6) δ 163.30, 157.89, 139.07, 138.38, 134.35, 129.33, 128.44, 128.36, 127.20, 126.86, 125.35, 124.80, 121.10, 119.82, 119.11, 71.69, 29.38. HRMS (ESI) calcd for $C_{19}H_{17}NNaO_3$ $[M + Na]^+$: 330.1101, Found: 330.1108.

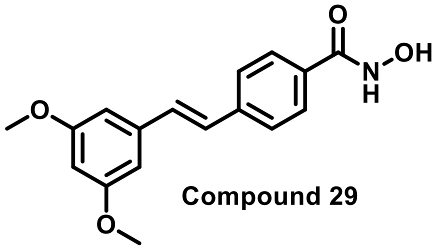

Compound 29

(E)-4-(3,5-dimethoxystyryl)-N-hydroxybenzamide (**29**)
White solid, Mp:149-150℃, Yield: 54.7%. ^{1}H NMR (400 MHz, DMSO-d_6) δ11.23 (s, 1H), 9.03(s, 1H), 7. 77(d, 2H, *J* = 8.4 Hz), 7.67 (d, 2H, *J* = 8.4 Hz), 7.33 (d, 1H, *J* = 16.4 Hz), 7.29 (d, 1H, *J* = 16.4 Hz), 6. 81(d, 2H, *J* = 2.4 Hz), 6.45 (t, 1H, *J* = 2.4 Hz), 3.79 (s, 6H). ^{13}C NMR (101 MHz, DMSO-d_6) δ 164.36, 161.15, 140.18, 139.24, 132.02, 130.69, 128.49, 127.81, 126.83, 105.17, 100.72, 55.70. HRMS (ESI) calcd for $C_{17}H_{16}NO_4$ $[M - H]^-$: 298.1085, Found: 298.1083.

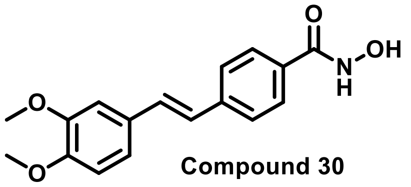

Compound 30

(E)-4-(3,4-dimethoxystyryl)-N-hydroxybenzamide (**30**)
White solid, Mp:197-198℃, Yield: 52.6%. ^{1}H NMR (400 MHz, DMSO-d_6) δ 11.23 (s, 1H), 9.05 (s, 1H), 7.77 (d, 2H, *J* = 8.4 Hz), 7.64 (d, 2H, *J* = 8.0 Hz),7.33-7.28 (m, 2H), 7.19 (d, 1H, *J* = 16.4 Hz), 7.14 (dd, 1H, J_1 = 1.6 Hz, J_2 = 8.4 Hz), 6.97 (d, 1H, *J* = 8.4 Hz), 3.84 (s, 3H), 3.78 (s, 3H). ^{13}C NMR (101 MHz, DMSO-d_6) δ 164.45, 149.54, 149.42, 140.71, 131.48, 130.71, 130.13, 127.80, 126.42, 125.76, 120.85, 112.20, 109.76, 55.97. HRMS (ESI) calcd for $C_{17}H_{16}NO_4$$[M - H]^-$: 298.1085, Found: 298.1087.

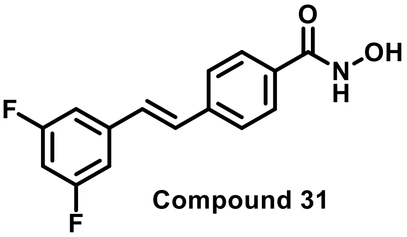

Compound 31

(E)-4-(3,5-difluorostyryl)-N-hydroxybenzamide (**31**)

White solid, Mp:266-267℃, Yield: 45.3%. ^{1}H NMR (400 MHz, DMSO-*d*$_6$) δ 11.27 (s, 1H), 9.08 (s, 1H), 7.80 (d, 2H, *J* = 8.4 Hz), 7.68 (d, 2H, *J* = 8.4 Hz), 7.48 (d, 1H, *J* = 16.4 Hz), 7.41-7.35 (m, 3H), 7.16 (t, 1H, *J* = 9.2 Hz). ^{13}C NMR (101 MHz, DMSO-*d*$_6$) δ 164.49, 164.35, 164.25, 162.05, 161.91, 141.38, 141.28, 141.18, 139.50, 132.63, 131.00, 128.44, 128.41, 128.38, 127.86, 127.15, 110.11, 110.04, 109.92, 109.85, 103.75, 103.49, 103.23. HRMS (ESI) calcd for $C_{15}H_{10}F_2NO_2$ $[M - H]^-$: 274.0685, Found: 274.0684.

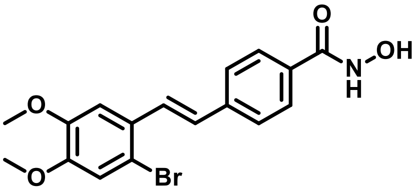

Compound 32

(E)-4-(2-bromo-4,5-dimethoxystyryl)-N-hydroxybenzamide (**32**)

White solid, Mp:163-164℃, Yield: 65.9%. ^{1}H NMR (400MHz, DMSO-*d*$_6$) δ 11.26 (s, 1H), 9.06 (s, 1H), 7.78 (d, 2H, *J* = 8.4 Hz),7.65 (d, H, *J* = 8.4 Hz), 7.41 (s, 1H), 7.39 (d, 1H, *J* = 15.2 Hz), 7.30 (d, 1H, *J* = 16.0 Hz), 7.20 (s, 1H), 3.86 (s, 3H), 3.81 (s, 3H). ^{13}C NMR (101 MHz, DMSO-*d*$_6$) δ 164.33, 150.09, 149.08, 140.11, 132.14, 129.30, 128.45, 128.28, 127.94, 126.75, 115.92, 114.80, 109.78, 56.39, 56.30. HRMS (ESI) calcd for $C_{17}H_{16}BrNNaO_4$ $[M + Na]^+$: 400.0155, Found: 400.0158.

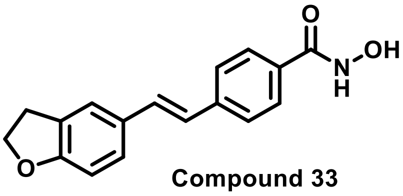

Compound 33

(E)-4-(2-(2,3-dihydrobenzofuran-5-yl)vinyl)-N-hydroxybenzamide (**33**)

Yellow solid, Mp:193-194℃, Yield: 50.1%. ^{1}H NMR (400MHz, DMSO-*d*$_6$) δ 12.20 (s, 1H), 9.01 (s, 1H), 7.74 (d, 2H, *J* = 8.4 Hz), 7.61 (d, 2H, *J* = 8.4 Hz), 7.56 (d, 1H, *J* = 1.6 Hz), 7.32 (dd, 1H, J_1 = 1.6, J_2 = 8.0 Hz), 7.30 (d, 1H, *J* = 16.4 Hz), 7.11 (d, 1H, *J* = 16.4 Hz), 6.78 (d, 1H, *J* = 8.0 Hz), 4.56 (t, 2H, *J* = 8.4 Hz), 3.21 (t, 2H, *J* = 8.4 Hz). ^{13}C NMR (101 MHz, DMSO-*d*$_6$) δ 164.42, 160.41, 140.80, 131.34, 130.76, 129.93, 128.58, 127.97, 127.75, 126.34, 124.96, 123.45, 109.54, 71.72, 29.32. HRMS (ESI) calcd for $C_{17}H_{14}NO_3$$[M - H]^-$: 280.0979, Found: 280.0980.

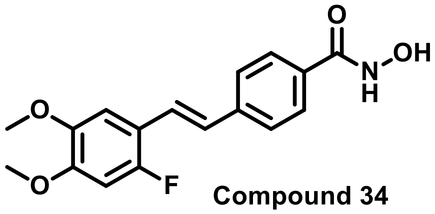

Compound 34

(E)-4-(2-fluoro-4,5-dimethoxystyryl)-N-hydroxybenzamide (**34**)
White solid, Mp:159-160℃, Yield: 71.6%.^{1}H NMR (400 MHz, DMSO-d_6) δ 11.24 (s, 1H), 9.04 (s, 1H), 7.77 (d, 2H, J = 8.0 Hz), 7.66 (d, 2H, J = 8.0 Hz), 7.36-7.26 (m, 3H), 6.95 (d, 1H, J = 12.4 Hz), 3.83 (s, 3H), 3.80 (s, 3H). ^{13}C NMR (101 MHz, DMSO-d_6) δ 164.38, 156.14, 153.74, 150.38, 150.28, 145.99, 145.97, 140.40, 131.88, 128.07, 128.03, 127.85, 126.62, 122.26, 115.66, 115.53, 109.50, 109.46, 101.14, 100.86, 56.56, 56.44, 40.38. HRMS (ESI) calcd for $C_{17}H_{15}FNO_4$ [M－H]$^-$: 316.0991, Found: 316.0989.

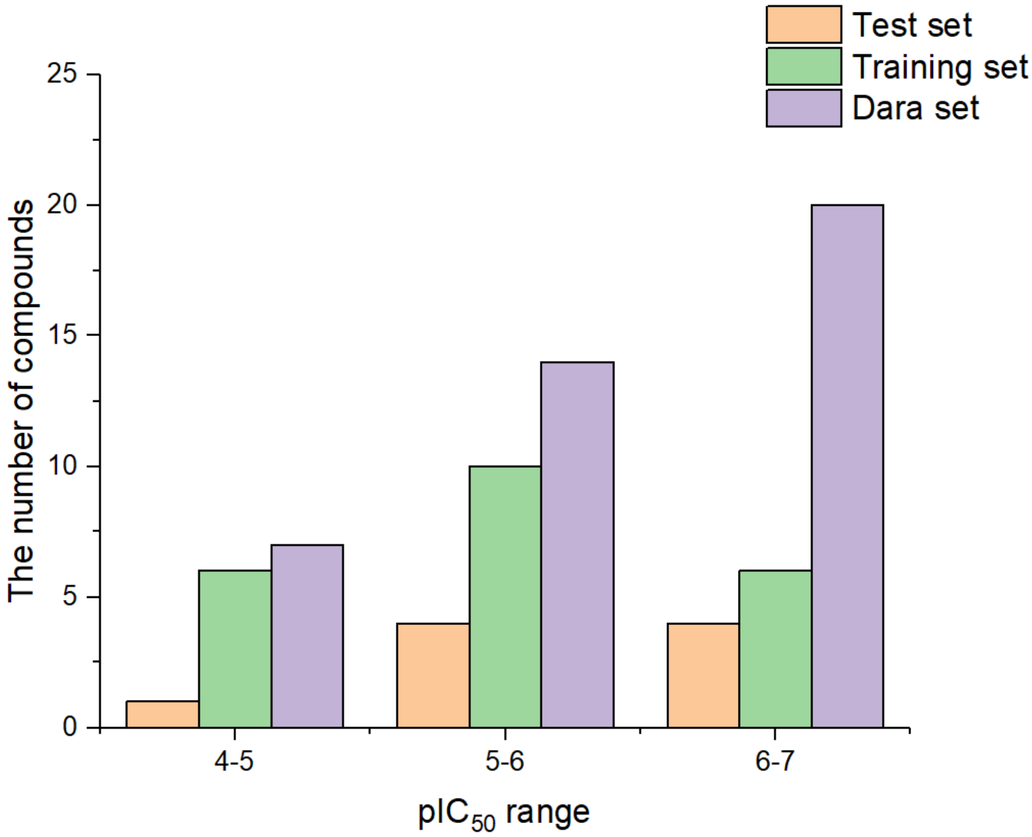

Figure S1. 3D-QSAR Histogram of activity data distribution

S2. MOE2015 docking process

Like Gilde, the crystal structure was processed before docking. Open the docking program. When docking in the FAD region, MOE2015 will automatically identify the FAD region as the docking site (click siteview to observe in the main display window). When docking the substrate region, in order to set the docking region correctly, we need to manually set the docking site. Receptor is chosen to be the Receptor atoms, the site is chosen to be the selected atoms, and then in the display window, region of histone H3 is defined as the docking site. (Unlike Glide, MOE2015 automatically generated pockets of appropriate size according to the central setting of the bind site, without setting the size of the box). Set placement poses = 20, refinement poses = 20. In the docking process, the docking energy of ligands in different conformations is obtained, including the electrostatic potential energy between protein and ligand, van der Waals energy, etc. the sum of these energy parameters represents the advantages and disadvantages of the docking results. After the above operations, 41 small molecules with minimal energy were docked in FAD-binding site and substrate-binding site, respectively. Each small molecule generated 20 conformations and preserved the pose of the first 10 GBVI/WSA dG score to predict the binding mode of these compounds.

Table S1. Randomizations of biological activity for the Y-random test.

Random_1	Random_2	Random_3	Random_4	Random_5	Random_6	Random_7	Random_8	Random_9	Random_10
6.036	6.308	5.285	5.888	5.373	6.548	6.917	4.443	4.443	5.447
6.05	4.443	6.408	5.889	5.447	6.439	6.91	6.154	6.154	5.373
6.066	4.529	6.439	6.036	5.48	6.408	6.717	6.143	6.143	5.331
6.088	4.696	6.548	6.05	5.587	6.308	6.678	6.117	6.117	6.548
6.117	4.722	6.678	6.066	5.833	6.16	5.285	6.088	6.088	6.439
6.143	4.79	6.717	6.088	6.16	6.05	5.044	6.066	6.066	6.408
6.154	4.991	6.91	6.117	6.308	6.036	4.991	6.548	5.044	6.308
6.16	5.044	6.917	6.143	6.408	5.889	4.79	6.439	4.991	6.917
6.308	5.285	5.331	6.154	6.439	5.888	4.722	6.408	4.79	6.91
4.443	6.408	5.373	4.696	6.548	5.833	4.696	6.308	4.722	6.717
4.529	6.439	5.447	4.722	6.678	5.587	4.529	6.917	4.696	6.678
4.696	6.548	5.48	4.79	6.717	5.48	4.443	6.91	4.529	5.285
4.722	6.678	5.587	4.991	6.91	5.447	6.154	6.717	6.16	4.991
4.79	6.717	5.833	5.044	6.917	5.373	6.143	6.678	6.05	4.79
4.991	6.91	5.888	5.285	4.443	5.331	6.117	5.285	6.036	4.722
5.044	6.917	5.889	5.331	4.529	6.917	6.088	5.044	5.889	4.696
5.285	5.331	6.036	5.373	5.888	6.91	6.066	4.991	5.888	4.529
6.408	5.373	6.05	5.447	5.889	6.717	6.548	4.79	5.833	6.16
6.439	5.447	6.066	5.48	6.036	6.678	6.439	4.722	5.587	6.05
6.548	5.48	6.088	5.587	6.05	5.285	6.408	4.696	5.48	6.036
6.678	5.587	6.117	5.833	6.066	5.044	6.308	4.529	5.447	5.889
6.717	5.833	6.143	6.16	6.088	4.991	6.16	6.16	5.373	5.888
6.91	5.888	6.154	6.308	6.117	4.79	6.05	6.05	5.331	5.833
6.917	5.889	6.16	6.408	6.143	4.722	6.036	6.036	6.548	5.587
5.331	6.036	6.308	6.439	6.154	4.696	5.889	5.889	6.439	5.48
5.373	6.05	4.443	6.548	4.696	4.529	5.888	5.888	6.408	4.443
5.447	6.066	4.529	6.678	4.722	4.443	5.833	5.833	6.308	6.154
5.48	6.088	4.696	6.717	4.79	6.154	5.587	5.587	6.917	6.143
5.587	6.117	4.722	6.91	4.991	6.143	5.48	5.48	6.91	6.117
5.833	6.143	4.79	6.917	5.044	6.117	5.447	5.447	6.717	6.088
5.888	6.154	4.991	4.443	5.285	6.088	5.373	5.373	6.678	6.066
5.889	6.16	5.044	4.529	5.331	6.066	5.331	5.331	5.285	5.044

S3. Structural validation

The Ramachandran plot obtained through the Procheck program was shown in Figure S2. The overall G-factor value of model is 0.02. The torsion angles phi (φ) and psi (ψ) distributions of the Ramachandran plot of all non-glycine and non-proline residues, as shown in Table S2.

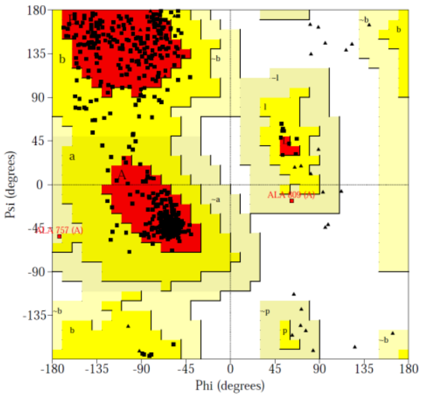

Figure S2. Ramachandran plot. Most favored (red), additionally allowed (yellow), generously allowed (pale yellow) and disallowed regions (white color).

It can be seen from Fig.S2 that most residues were in the most favored region, a good quality model would be expected to have over 90% in the most favoured regions. From Table S2, 91.2% residues were in most favoured regions, while 8.5% residues were in additional allowed regions, and only 0.3% residues were in generously allowed regions, no one residue was in disallowed regions. These results showed that the model had good stereo-chemical quality.

Table S2. Residues falling in the core region of the Ramachandran's plot.

Residues in most favoured regions	91.2%
Residues in additional allowed regions	8.5%
Residues in generously allowed regions	0.3%
Residues in disallowed regions	0.0%
Number of non-glycine and non-proline residues	100.0%

At the same time, verify 3D and ERRAT were used to evaluate the quality of the model structure. Verify 3D judged the reliability of the model by comparing its 3D profile with its sequence. It is generally believed that at least 80% residues of the models with average 3D-1D score ≥ 0.2 are acceptable. According to Fig.S3, 85.36% residues had an average 3D-1D score ≥ 0.2, indicated that the structure had a good environment profile.

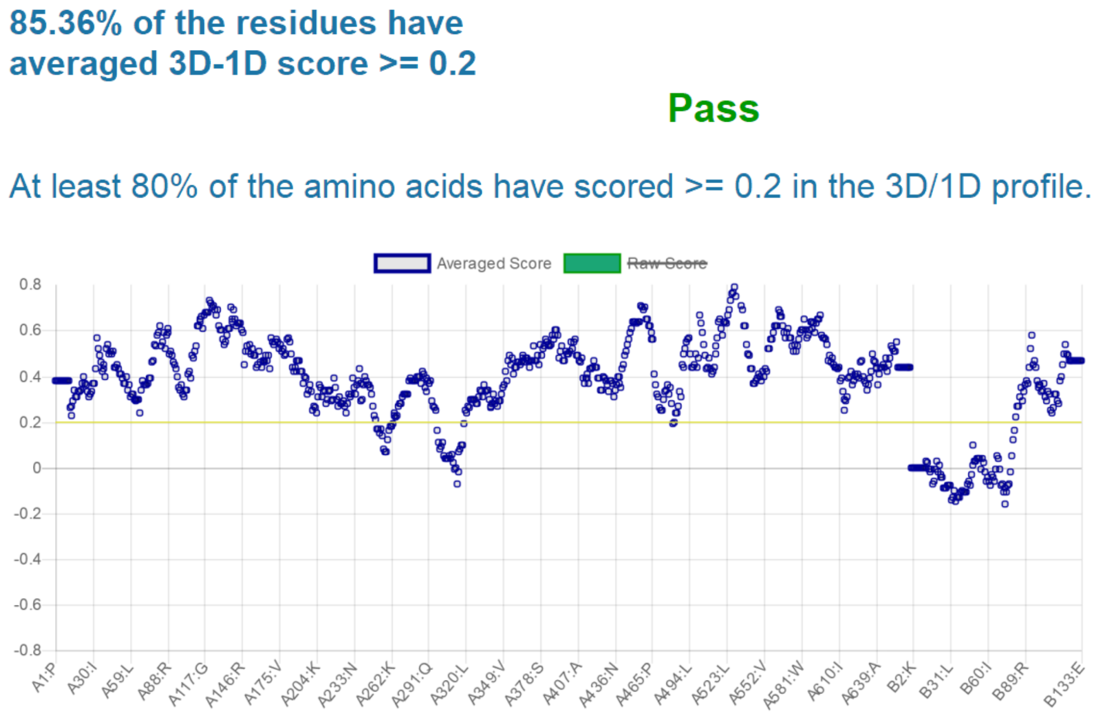

Figure S3. Verify 3D plots for model.

ERRAT evaluates the reliability of existing structures by comparing them with other high-precision structures. From Fig.S4, the overall quality factors of chain A and chain B of the model were 90.229 and 89.516, respectively, which mean that 90.229% residues in chain A were lower than the rejection limit of 95%. 89.516% residues in chain B were lower than the rejection limit of 95%. All the above evidences showed that the model used in this study had

good quality and can be used in molecular docking and MD research.

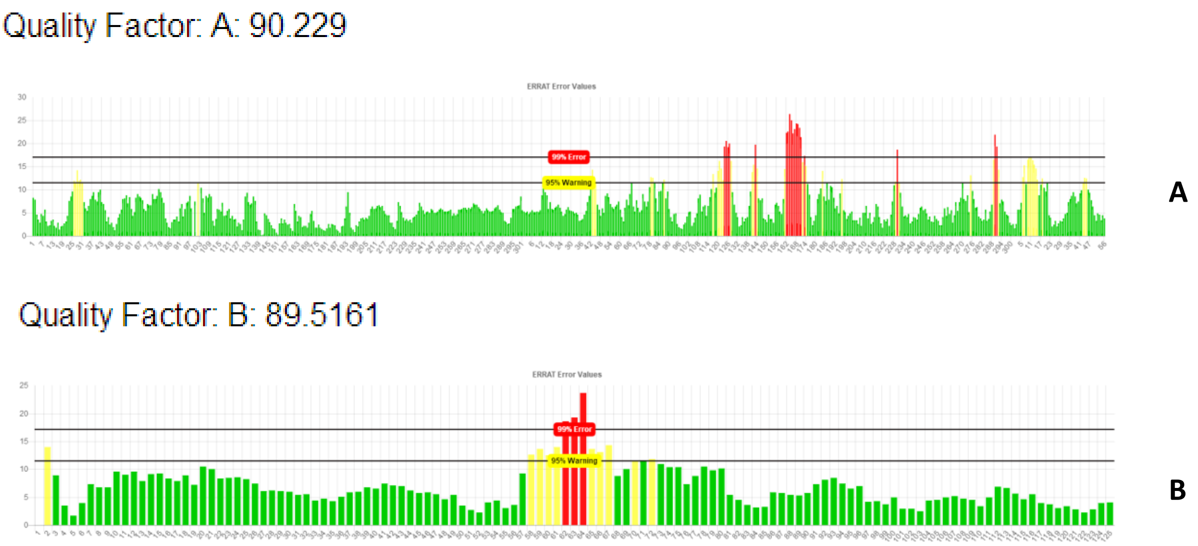

Figure S4. ERRAT result for model. (A) Chain A in 2V1D (LSD1). (B) Chain B in 2V1D (CoREST)

All the above evidences showed that the model used in this study had good quality and can be used in molecular docking and MD research.

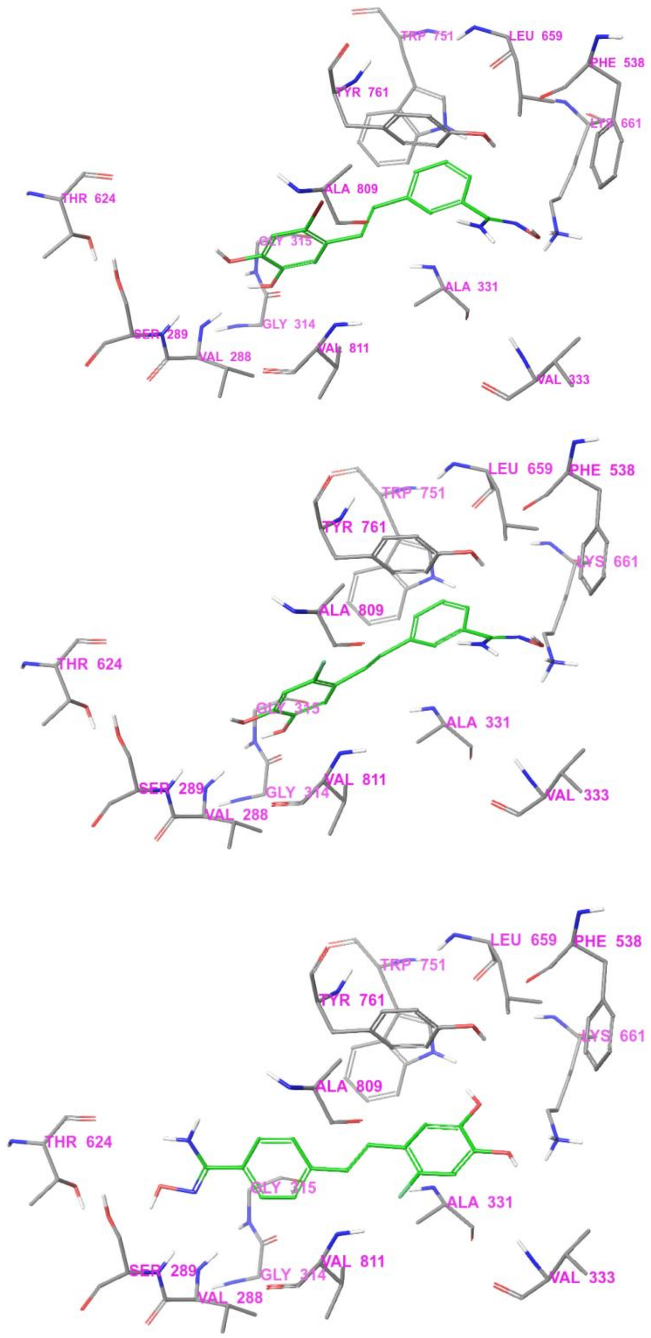

Figure S5. Docking results of the compound 04(A), 05(B) and 10(C) under type B. The carbon atoms of compound are shown in green. The nitrogen, oxygen, fluorine and bromine atoms were shown in blue, red, green and brown, respectively.

Table S3. MOE2015 docking results. The accounts of type A and type B in top 10 compounds, and the type of the highest score conformation.

No.	Tepy A	Tepy B	Best Score
1	1	7	B:-7.5274
2	0	6	B:-7.4051
3	2	3	-7.7093
4	3	6	A:-7.9427
5	1	7	B:-7.6839
6	1	4	-7.3688
7	5	5	A:-7.3687
8	8	1	A:-7.2838
9	5	2	-7.2838
10	3	7	B:-7.4012
11	9	1	A:-7.9820
12	3	2	A:-6.7127
13	4	1	A:-8.1098
14	6	4	A:-8.8715
15	8	2	A:-8.6284
16	10	0	A:-8.8072
17	4	6	A:-8.283
18	2	3	A:-8.1705
19	1	4	-8.5143
20	6	4	A:-8.9361
21	8	2	A:-8.9086
22	4	3	A:-9.1439
23	5	4	A:-8.2960
24	9	1	A:-8.9277
25	10	0	A:-8.9620
26	10	0	A:-8.7338
27	10	0	A:-9.4171
28	8	2	B:-8.5176
29	9	0	A:-8.5305
30	9	1	A:-8.3853
31	8	0	A:-7.5106
32	10	0	A:-8.5325
33	9	1	A:-7.9635
34	9	1	A:-8.2327
35	5	3	A:-7.5635
36	6	4	A:-7.3431
37	9	0	-7.9563
38	6	2	A:-8.3826
39	3	7	B:-9.5128
40	6	4	A:-9.0582

41	4	6	A:-8.4531

In Best score, if no label belongs to type A or B, the conformation of Top1 score belongs to other orientations, which rarely occurs (such as compound 3, 6, 9, 19, 37), indicating that these orientations are not the most likely orientation. So did not record.

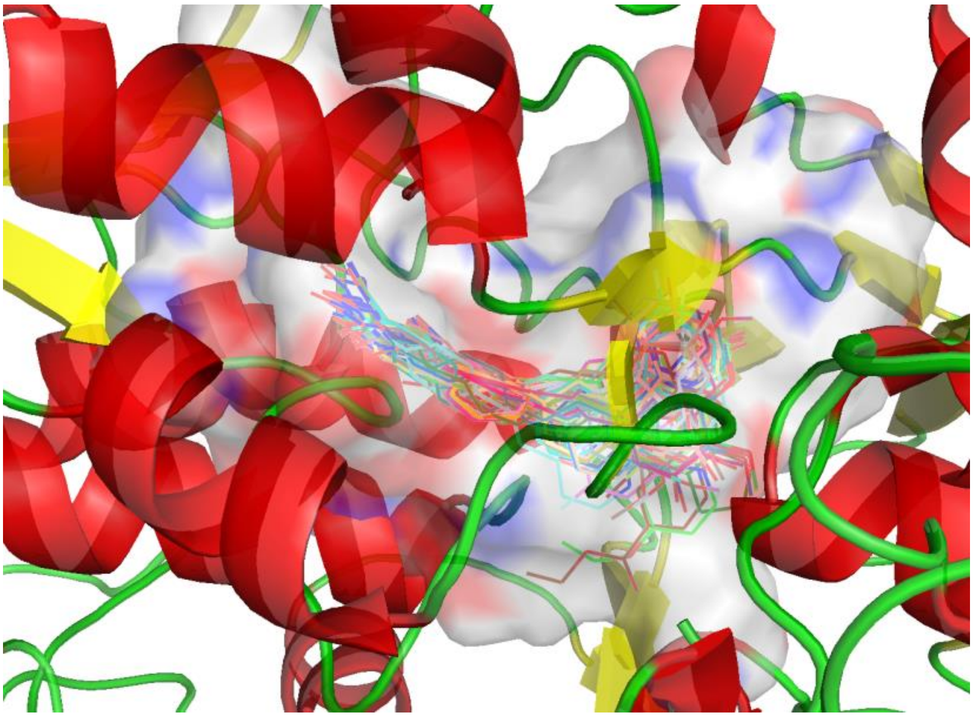

Figure S6. All compounds were docked into the FAD-binding site. The α helix structure was shown in red. The β sheet structure was shown in yellow. The Coil structure was represented in green.

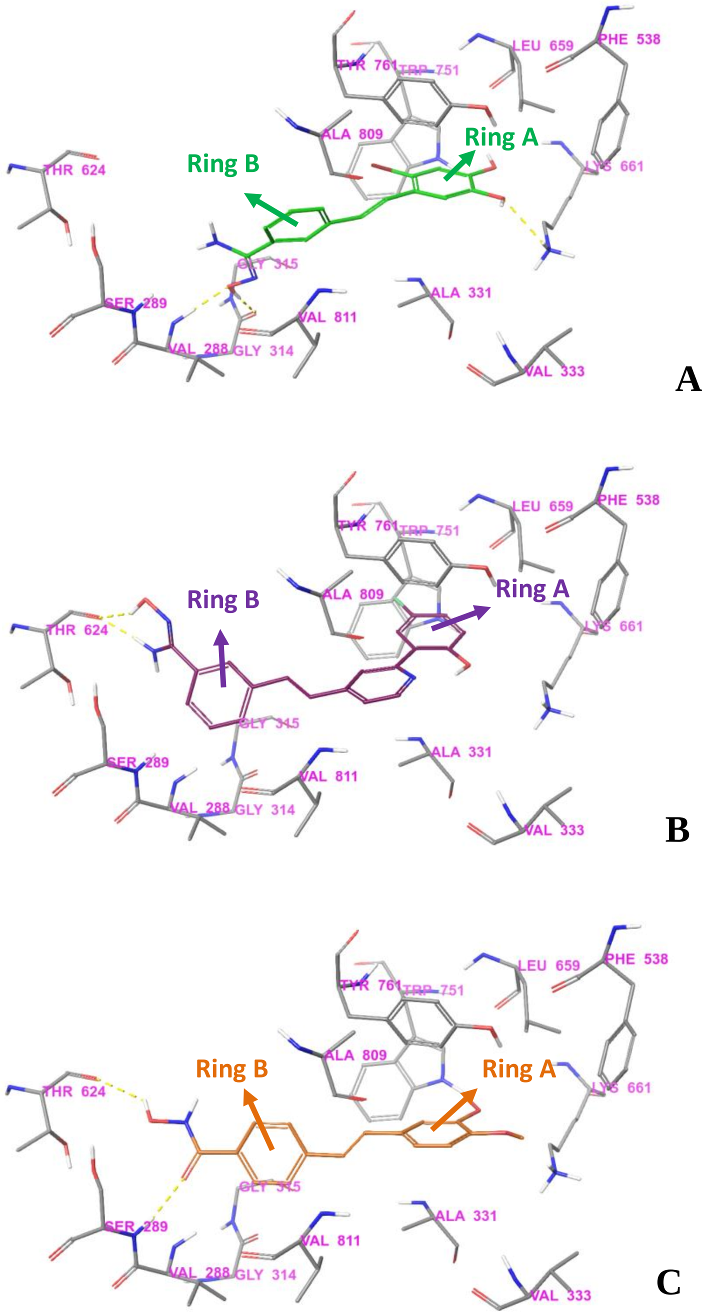

Figure S7. Docking results of the compound 04(A), 22(B) and 29(C) with LSD1. The carbon atoms of compound 04, 22 and 29 were shown in green, purple and orange, respectively. The nitrogen, oxygen, chlorine and bromine atoms were shown in blue, red, green and brown, respectively. H-bonds were shown in yellow dash lines.

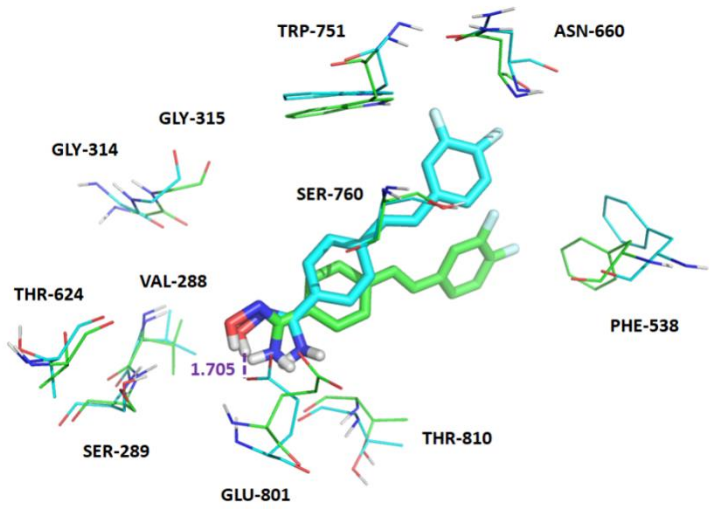

Figure S8. The superposition of the docking structure and structure of compound 35 in 36ns MD. Carbon atoms of docking result and 36ns MD structure were shown in green and cyan, respectively. H-bond of docking result and MD result were shown in red dash line and purple dash line, respectively.

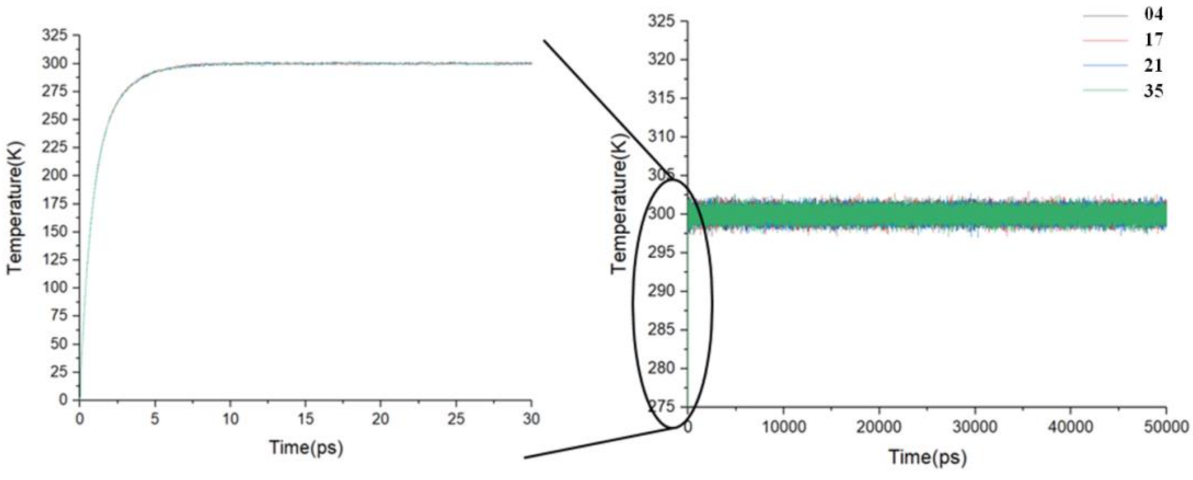

Figure S9. Temperature fluctuation plot in MD.
